# Validating and comparing reported indoor photovoltaic performance

**DOI:** 10.1039/d6el00150e

**Published:** 2026-10-27

**Authors:** Austin M. Kay, Gregory Burwell, Drew B. Riley, Paul Meredith

**Affiliations:** a Centre for Integrative Semiconductor Materials (CISM) and Department of Physics, Swansea University Bay Campus Swansea SA1 8EN UK A.M.Kay@Swansea.ac.uk Paul.Meredith@Swansea.ac.uk

## Abstract

Indoor photovoltaics (PVs) have garnered significant attention in recent years as promising power sources for wireless Internet-of-Things devices. However, the use of unstandardised testing practices makes it difficult to compare reported performance metrics. Discrepancies between experimental apparatus and methodologies can arise in a number of ways, including non-standard light sources with varying spectral irradiance (irrespective of the colour temperature or the colour-rendering index), spectral deviations induced during light attenuation, spatial non-uniformities, and temporal instability and an angular distribution of the light source. Unstandardised practices lead to variations in the spectral shape and light power impinging on a PV device, resulting in variations in the output current and power conversion efficiency. In this contribution, we explore the imprecision that arises when comparing previously reported unstandardised measurements. By quantifying and combining different sources of measurement imprecision, retrospective thermodynamic analyses are used to validate reported measurements for a variety of established and next-generation PV systems. These retrospective analyses allow each measurement to be categorised as either thermodynamically valid, plausible, or implausible. Ultimately, our investigations clarify the current upper bounds of state-of-the-art indoor PV performance, with the maximum validated power conversion efficiencies of the indoor PVs we consider being 40.3%, 29.2%, and 40.9% for perovskite, organic, and inorganic semiconductor-based systems, respectively.

## Abstract 2

Examination of indoor photovoltaic (IPV) testing apparatus reveals several imprecisions when comparing unstandardised measurements. In light of this, retrospective thermodynamic analyses are used to validate state-of-the-art IPV performance.

## Graphical abstract

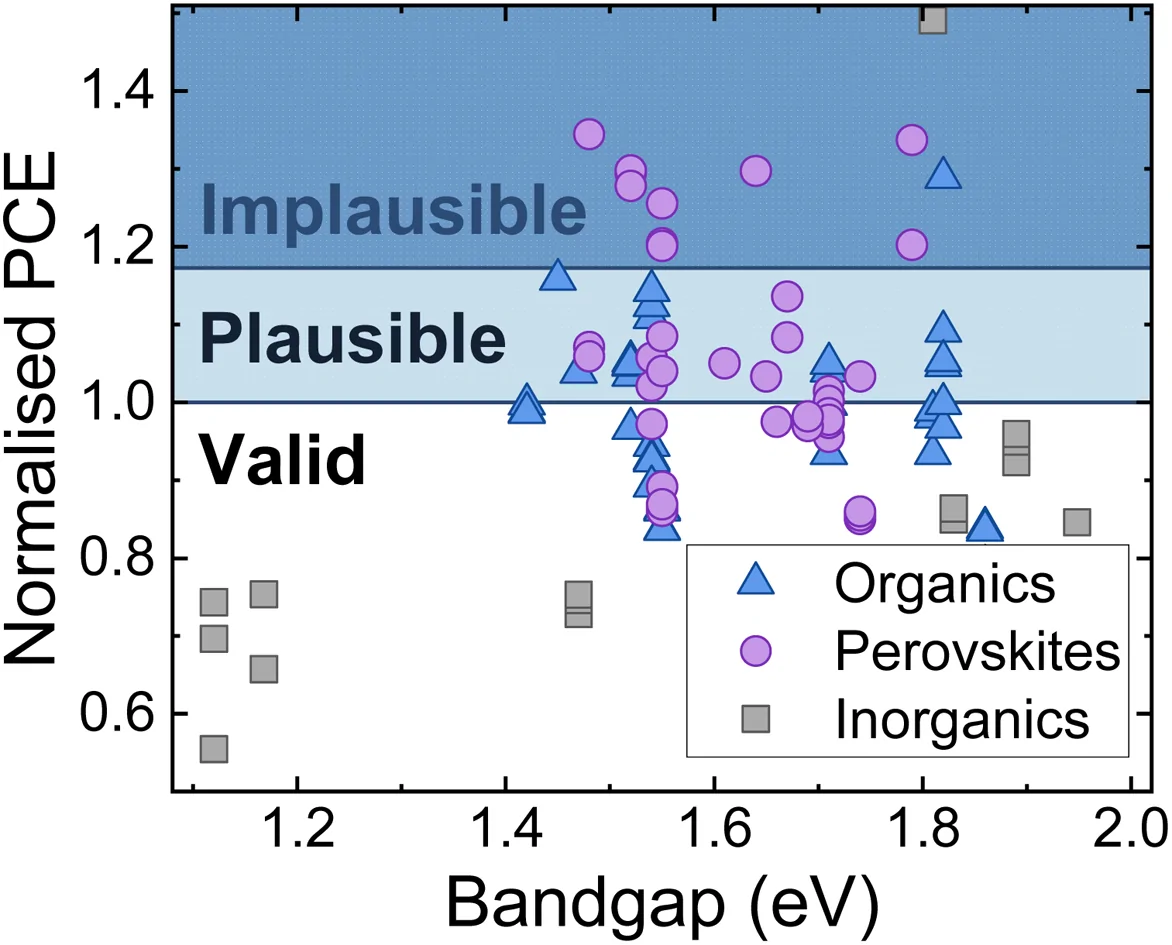

**Broader context**

Indoor photovoltaics (PV) are devices that harvest indoor light to generate local clean energy. They are strong candidates to power the billions of internet of things devices being rapidly deployed, which would otherwise need replaceable batteries or hard-wiring. Indoor PV is thus a rapidly emerging field, and next-generation semiconductors such as organics and perovskites are particularly suited to the application because of their tuneable bandgaps and intrinsically low embodied energy. As is common in the early stages of a high-tech device field, there are no agreed pre-standard research measurement protocols to allow accurate and reliable inter-comparison between different technologies, often reported in high-impact literature as state-of-the-art or new world records. In our paper we address this issue. We define the inconsistencies arising from different methodologies, and we demonstrate a first-principles thermodynamic and electro-optical model that can calculate the efficiency of any PV device under arbitrary illumination from the published AM1.5G performance and external quantum efficiency. We survey a large number of published results and classify them as either valid, plausible, or implausible. This approach allows us to predict maximum efficiencies and is a means by which state-of-the-art results can be validated in the absence of agreed measurement protocols.

## 1. Introduction

In late 2025 the number of Internet-of-Things (IoT) devices deployed across the globe grew to more than 20 billion – a figure that is anticipated to double by 2030.^1^ While the combined energy consumption of all IoT devices has grown sharply in recent years, the operational power requirements of individual IoT devices have, in fact, decreased due to continuous improvements in circuit design and wireless transmission rates (which reduce the duration of the devices' higher-current ‘active mode’).^2^ Depending on the communication protocol,^3^ modern IoT devices require as little as micro-watts of power to operate.^4,5^ Such low power requirements can be met sustainably through energy-harvesting techniques, including piezo-electric,^6,7^ thermoelectric,^8,9^ and microbial-based methods,^10^ which replace disposable batteries and reduce maintenance costs by supplying IoT devices with locally generated power. One particularly promising approach for sustainably powering IoT devices deployed in homes, workspaces, and other internal places are indoor photovoltaics (PVs), which convert low-intensity ambient light into electrical power.^11–14^

Over the last decade, the number of scientific publications related to indoor PVs has mirrored the recent proliferation of IoT devices. These publications encompass a diverse range of PV materials, ranging from solution-processible and spectrally tuneable perovskite,^15–18^ organic,^19,20^ and dye-sensitised PVs,^21,22^ to more conventional inorganic PVs.^13,23^ However, due to the widespread use of unstandardised measurement protocols, intra- and inter-material and system comparisons of the power conversion efficiencies (PCEs) of indoor PVs are often infeasible. The principal experimental factors that lead to imprecision when comparing values reported without adhering to any standardised testing protocol include spectral variations between light sources, spectrally modifying attenuation techniques, geometrically induced spatial non-uniformities, and varying angular distributions (collimated *versus* diffuse) and temporal instabilities in the irradiance emitted by light sources.^24–26^ As the spectral overlap between the response of an indoor PV and the irradiance will differ between light sources, characterisation using different sources can lead to vastly different PCEs, even when two sources possess the same correlated colour temperature (CCT).^27^ Further exacerbating this issue, spectral uncertainties can be introduced when attenuating the light source to typical indoor irradiances, as common attenuation techniques often modify the irradiance spectrum.^25,28^ Similar spectral uncertainties can be introduced throughout the course of an experiment as the irradiance of a light source may vary due to fluctuations in temperature and current. Other, non-spectral factors include spatial non-uniformity (SNU) in the irradiance across an indoor PV device-under-test, which can produce non-uniform charge densities and localised heating, leading to increased uncertainty in the resulting metrics, especially for larger-area devices.^28^ Likewise, the angular distribution of light impinging on the imaging plane – coupled with differences between the angular responses of a standardised reference cell (used to fix the light intensity) and an indoor PV device-under-test – can introduce further measurement uncertainties.

As with all precision technology, creation and wide-spread adoption of standardised testing protocols is mandatory to alleviate many of the difficulties in comparing and benchmarking indoor PVs. In turn, this will not only enable robust comparisons between reported literature values but will also serve to generate confidence in – and allow a genuine industrial base to develop around – indoor PVs as power sources for IoT devices. An analogous example would be the adoption of the AM1.5G standard for solar-harvesting PVs, which was fundamental in developing assurances in terrestrial PVs and ultimately led to increased consumer confidence, globally trusted supply chains, and industrial development of emerging technologies. In this contribution we remain agnostic as to the technical details of any such standard, as many have been proposed,^29–31^ and because this will ultimately be determined by the International Electrotechnical Commission (IEC). We do however emphasise the requirement for one such standard protocol and suggest that the relevant journals adopt standardised reporting summaries, as is done for terrestrial PVs.^32^ A nascent testing standard published by the IEC, which we utilise in this work, specifies many factors needed to accurately characterise indoor PVs.^31^ This standard (IEC TS 62607-7-2:2023) addresses some of the experimental factors described above by including two ideal standard light sources (CIE LED-B4 and CIE FL-10), a spectral mismatch calculation to account for variations in source spectrum, an agreed-upon illuminance range, and ratings for spatial and temporal stability of the sources. This standard does not, however, make any recommendation with respect to the angular distribution of the light. As many indoor PV reports have been published without adhering to this or any other testing standard, the reported performance values cannot form the basis of a robust intercomparison.

Motivated thus, in this work we consider the various experimental factors that can lead to imprecision when comparing the reported PCEs of indoor PVs, and assign minimum values to the uncertainties associated with these factors. We then perform retrospective thermodynamic analyses on reported indoor PV performance from a wide variety of literature sources. In these analyses we collapse the reported values onto the IEC standard, however, this retrospective framework could be applied with respect to any standard. Furthermore, these thermodynamic analyses assume well-defined light sources and ideal cell conditions before the uncertainties described above are applied. We evaluate the real experimental uncertainty associated with the use of diffuse *versus* collimated light sources and find that the use of pseudo-collimated light sources over collimated ones can lead to at least 3% uncertainty in the PCE. The results are, in some cases, that the reported experimental metrics surpass thermodynamic limits, even after accounting for the expected levels of imprecision associated with comparing results *via* the IEC standard. By considering the thermodynamic plausibility of many non-standardised measurements, reported state-of-the-art indoor PV performance is re-assessed. At irradiances around 0.3 mW cm^−2^ (or illuminances around 1000 lux), the maximum validated PCEs of perovskite, organic, and inorganic semiconductor-based indoor PVs are clarified as 40.3%, 29.2%, and 40.9%, respectively. We contend that these figures should be considered as realistic upper bounds for the systems considered in our analysis, and may also be indicative of the broader material classes represented.

## 2. Quantifying comparison imprecisions in indoor photovoltaic characterisation

### 2.1. An overview on experimental sources of comparison imprecision

An illustration of a typical indoor PV testing apparatus is shown in Fig. 1a. The light source at the top of the apparatus irradiates in the direction prescribed by the imaging system, this light then propagates through an attenuator (to reduce the irradiance) before impinging on the device-under-test (DUT). If different reports used identical apparatus, comparison between measurements would be trivial. However, uncertainties can arise in each component of the apparatus.

**Fig. 1:**
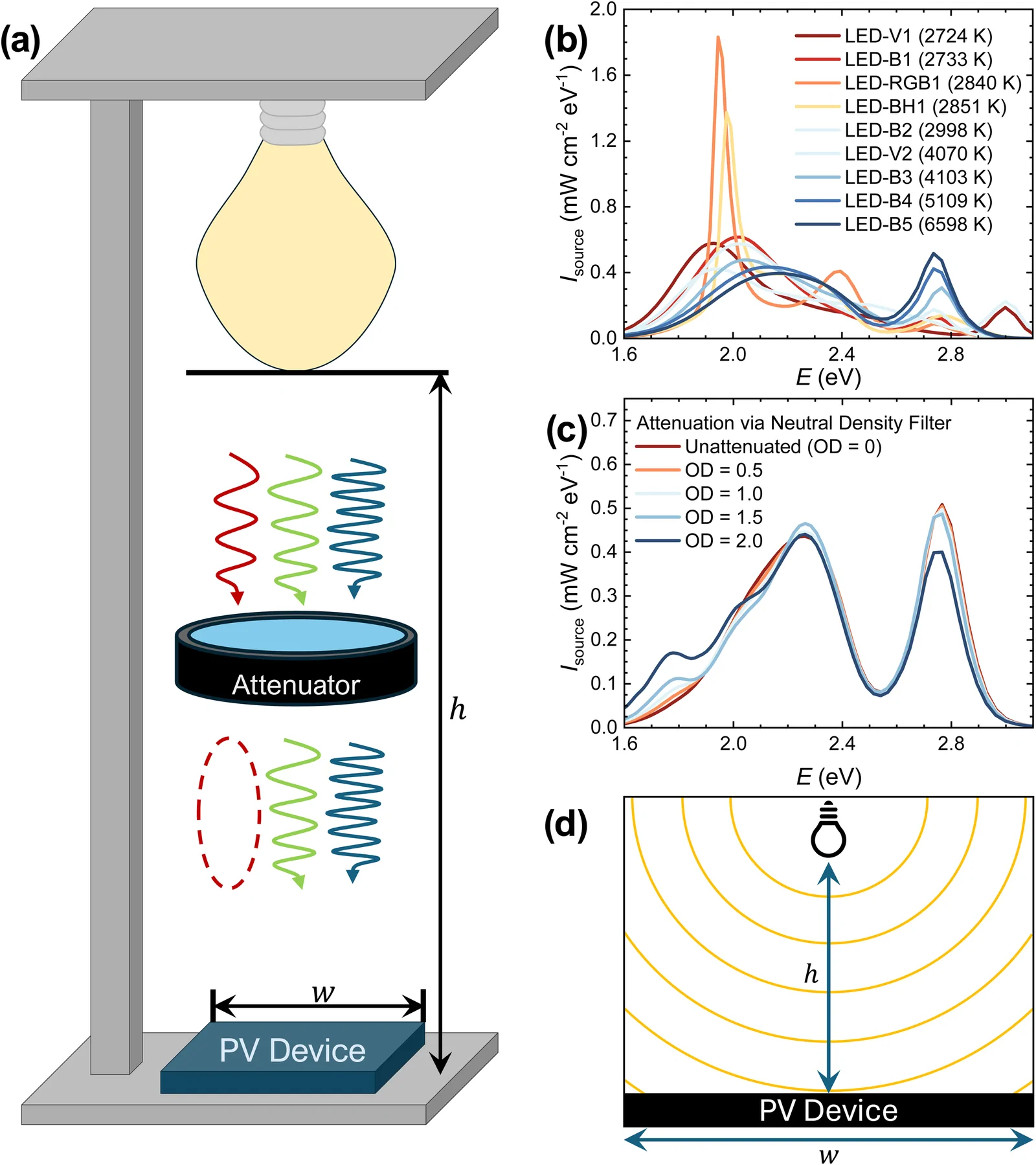
Exploring sources of experimental uncertainty in indoor photovoltaic (PV) characterisation. (a) Illustration of an indoor PV device-under-test (DUT) of width *w* being characterised using a typical setup, where the irradiance from the light source a distance *h* away is passed through an attenuator. (b) The spectral irradiances (*I*_source_) of International Commission on Illumination (CIE) light-emitting diodes (LEDs) plotted *versus* photon energy (*E*), where the correlated colour temperatures (CCTs) of each light source are indicated in brackets.^37^ Note that all spectra have integrated irradiance 
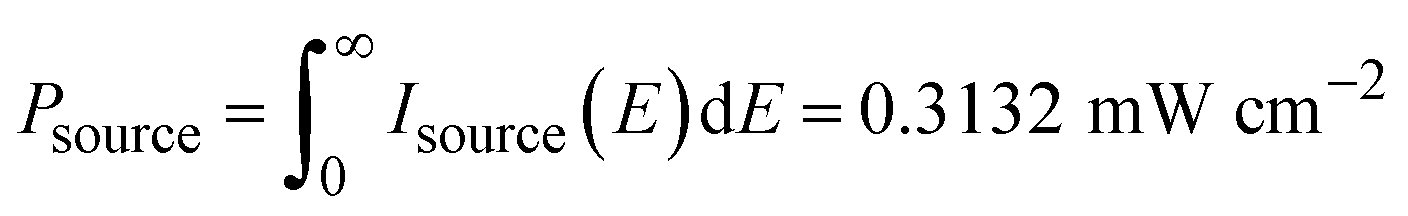
, corresponding to an illuminance of 1000 lux under LED-B4 illumination. (c) Deviation in the spectral irradiance of a 4000 K LED due to light attenuation by a neutral density filter with various optical densities (ODs).^28^ Once again, each of these spectra is scaled to *P*_source_ = 0.3132 mW cm^−2^. (d) Illustration of how the height (*h*) of a point source above a DUT influences the spatial non-uniformity (SNU) of the irradiance across it, where the concentric circular lines indicate contours in irradiance. For *h* ≃ *w*, the DUT cuts across several contour lines, producing large SNUs in irradiance across the device; for *h* ≫ *w*, on the other hand, SNUs are reduced as the device effectively traverses a contour line.

Starting at the top of the apparatus, different light sources often have unique spectral irradiances that – when overlapped with an indoor PV's spectral response – will result in differing PCEs. This is particularly non-trivial when comparing performance under different light-emitting diodes (LEDs), fluorescent lamps (FLs), halogens, or incandescent bulbs, as the spectral responses can vary significantly, especially in the near infrared or ultraviolet portions of the spectrum.^33^ Further uncertainties are introduced if the photometric units of illuminance are used (which accounts for the standard luminous efficiency function,^34^ also known as the photopic response of the human eye) instead of the radiometric units of irradiance, as the illuminance may not account for the additional radiation near the infrared or ultraviolet portions of the spectrum.^24,33^ In this work, light intensities are therefore specified in the radiometric units of irradiance, with the corresponding illuminances being provided only for comparison. Fig. 1b shows the spectral irradiances (*I*_source_) of exemplary International Commission on Illumination (CIE) standard LEDs, which are plotted *versus* photon energy (*E*) and are representative of the actual photon flux across the spectrum. For each curve the integrated irradiance (*P*_source_), defined by1
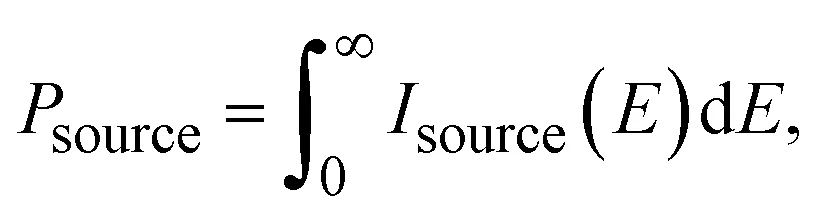
has been scaled to 0.3132 mW cm^−2^, which corresponds to an illuminance of 1000 lux under LED-B4 illumination. Note, however, that these standard LED irradiance spectra are somewhat idealised. In practice, light sources produced by different manufacturers can have different spectral irradiances, even when they have the same CCT; this important point is explored further in Section 2.2. However, it is important to note that experimentalists can correct for small variations in the spectrum of a light source through a spectral mismatch calculation and hence perfectly identical light sources are not required for robust comparisons. Despite this, many publications reporting indoor PV performance have either not reported sufficient spectral information or have not implemented a spectral mismatch calculation, preventing this correction from being reconstructed retrospectively. Consequently, in this work the contribution arising from spectral variation is retained in the retrospective validation framework. Such variations in a light source's irradiance spectrum may also be temporally dependent, due to fluctuations in driving current and temperature, as discussed in Section 2.3.

As indoor PVs are expected to operate across various levels of illumination, the IEC testing standard requires performance to be evaluated at illuminances ranging from 50 to 1000 lux.^31^ To achieve a desired light intensity at the plane of an indoor PV DUT, attenuation techniques must be used. Ideally, these techniques should be spectrally invariant across all light intensities; however, many attenuation techniques possess an intensity-dependent, wavelength-selective transmittance (or, in the case of mirror-based systems, a wavelength-selective reflectance), which modifies the lineshape of the spectral irradiance. This effect is demonstrated in Fig. 1c by the spectral irradiance of a 4000 K LED transmitted through neutral density filters with differing optical densities (ODs). In this panel, all spectra have been scaled to an integrated irradiance *P*_source_ = 0.3132 mW cm^−2^ using eqn (1) to facilitate lineshape comparisons. Clearly, the choice of light attenuation technique can alter the spectral irradiance across a DUT (relative to that of a light source) and, as explored in Section 2.4, this leads to additional imprecision when comparing reported indoor PV performance.

Additional, non-spectral factors can also affect the irradiance across the DUT, including spatial non-uniformity (SNU) and an angular distribution in the light relative to the imaging plane of the DUT. In basic apparatus (*i.e.*, those devoid of additional focusing optics) the geometric SNU is mainly determined by the size of the device relative to the distance from the light source, as illustrated by the example of a point source in Fig. 1d. An apparatus characterising a commercial-scale device with a width (*w*) of 5 cm would require a larger height (*h*) to maintain a low SNU compared to a laboratory scale-device with *w* < 1 cm. The effects of SNUs are explored further in Section 2.5. Like SNUs, an angular distribution in the light on a DUT can also impact its power output. For a perfectly collimated light source, all photons arrive at a single angle relative to the plane of the DUT (most investigations consider normal incidence only); for a perfectly diffuse light source, on the other hand, photons will impinge on the DUT equally from all incident angles. In practice, most indoor light sources will have angular distributions between these extremes, with a recent inter-laboratory comparison reporting angular distributions of ±10° for pseudo-collimated light sources.^35^ As the angular response of any PV cell will depend on the refractive indices of its constituent materials – and will therefore differ from one DUT to another – the degree of collimation of the light across the DUT may impact the reported PCE, as explored further in Section 2.6.

By following the same, top-down arrangement shown in Fig. 1, we will now work through each of these experimental factors in detail, assigning a realistic imprecision for comparisons between apparatus before validating reported indoor PV cells using retrospective thermodynamic analyses. To support these analyses, we have upgraded our open-source computational tool, *PV-Simulator*, with capabilities for validating measurements of indoor photovoltaics.^36^ This tool holds spectral data for more than a hundred sources of artificial light, alongside material data for several hundred photovoltaic devices, including the absorbance spectra, the one-Sun PV figures-of-merit, and – for many systems – the PV figures-of-merit under indoor illumination conditions.

### 2.2. Variation in spectral irradiance

Starting at the top of the apparatus shown in Fig. 1, the first experimental factor we consider is variation between the spectral irradiances of different light sources. As mentioned above, there will inevitably be spectral variations between light sources from different manufacturers, even in sources of equivalent CCT, which will result in some variation when comparing performance reported across different publications. Such variations are illustrated in Fig. 2a, where the spectral irradiances of several 3000 K LEDs reported in various scientific publications are plotted alongside the spectral irradiances of commercially available (CREE LED) 3000 K LEDs.^38–45^ Many of these LEDs have differing colour-rendering indices, which quantifies the fidelity with which the LEDs reveal the true colours of objects compared to sunlight. From Fig. 2a it is evident that the relative intensities and positions of the emission peaks of different LEDs can vary, despite each having the same CCT and being scaled to the same integrated irradiance. To explore how these spectral variations affect power generation in indoor PVs, we now consider three example systems whose photovoltaic external quantum efficiency (EQE_PV_) spectra are plotted *versus* photon energy in Fig. 2b. These systems were selected because their reported PCEs are close to apparent state-of-the-art and they exhibit similar energy gaps. As this analysis is intended to guide the field towards standardised approaches rather than single out specific sub-fields or erroneous publications, the three systems are simply labelled herein as “System A”, “System B”, and “System C”, and the references for these data have been purposefully omitted. These references will, however, be made available upon request from the corresponding authors. For clarity, System A is a perovskite-based indoor PV, whereas the other two are both organic-based indoor PVs; at around 1000 lux, these three systems exhibit PCEs of 40.0%, 31.2%, and 30.0%, respectively.

**Fig. 2:**
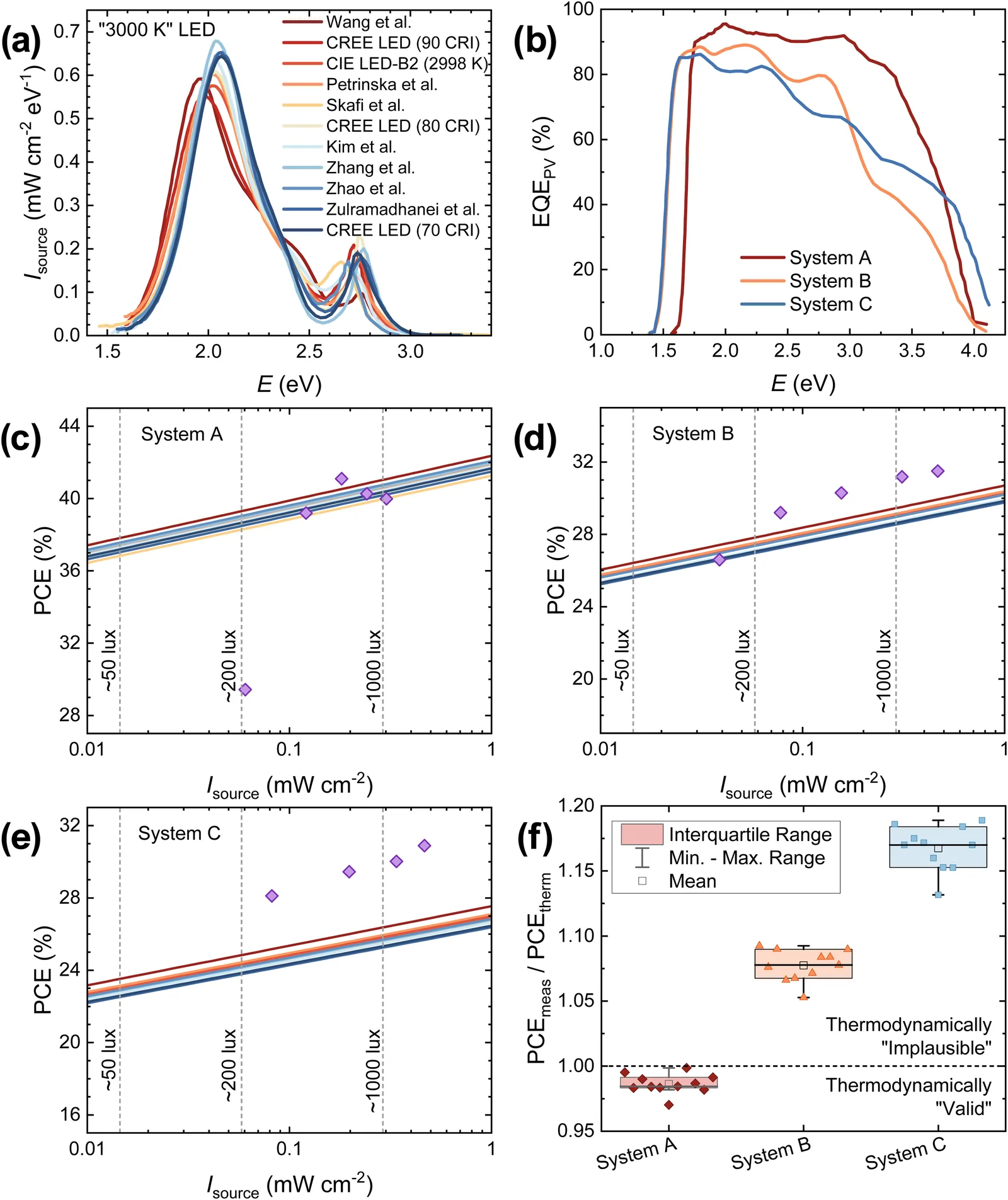
Comparing the effect of different “3000 K” LED spectral irradiances on the power output of indoor photovoltaics (PVs) simulated using thermodynamic device models.^46^ (a) Spectral irradiances (*I*_source_) of several 3000 K LEDs from different scientific publications and manufacturers plotted *versus* photon energy (*E*), where each source has *P*_source_ = 0.3132 mW cm^−2^.^38–45^ (b) Experimentally determined photovoltaic external quantum efficiency (EQE_PV_) spectra of three exemplary indoor PV systems, which are labelled as System A, B, and C. (c–e) Thermodynamic limit of the power conversion efficiencies (PCEs) of these indoor PV systems calculated using their EQE_PV_ spectra shown in (b) and the irradiance spectra shown in (a). In each of these panels, experimentally determined data are indicated by the purple diamonds. (f) For each of these systems, the PCEs measured experimentally (PCE_meas_ – filled symbols) at an irradiance *P*_source_ ∼ 0.3 mW cm^−2^ were normalised to the PCEs simulated in the thermodynamic limit (PCE_therm_) for all the different light sources in panel (a), allowing two classifications to be made: ‘thermodynamically valid’ – where PCE_meas_ ≤ PCE_therm_ for at least one of the light sources, and ‘thermodynamically implausible’ – where PCE_meas_ > PCE_therm_ for all light sources. The resultant spread in data across the different light sources are indicated by the box and whisker plots.

By combining the reported EQE_PV_ spectrum of each system with each irradiance spectrum from Fig. 2a, their power outputs could be determined in the thermodynamic limit.^46^ As described in SI Note 1, this model accounts for radiative open-circuit voltage losses induced by energetic disorder and sub-gap absorption, as well as the failure of the reciprocity principle for states that lie well below the gap.^47^ In addition, non-radiative open-circuit voltage losses under any illumination condition are estimated using a technique that compares the open-circuit voltage measured under one-sun illumination (
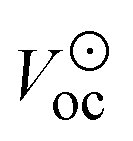
) with the corresponding open-circuit voltage expected from EQE_PV_ in the radiative limit.^47^ Using this approach, the thermodynamic limit of the PCE of the three indoor PV systems was calculated under the various 3000 K LED light sources. Note that the radiometric units of irradiance were used in these calculations to avoid further uncertainties arising from the photometric units of illuminance.^24^ Furthermore, an infinitely large shunt resistance was assumed, resulting in the highest-possible PCEs under indoor illumination conditions; *i.e.* this analysis provides a realistic ‘upper bound’ for the power output of the indoor PVs. Fig. 2c–e shows the results of these calculations *versus* the irradiance of the light source, where spectrally invariant attenuation has been used. Each line corresponds to a different source in Fig. 2a, where the line colours are the same. The experimentally reported PCEs for each system are shown as purple diamonds in each panel.

From the differences between the simulated curves in Fig. 2c–e, it is readily apparent that variations in the overlap between each system's EQE_PV_ spectrum and the spectral irradiances of the different 3000 K LEDs cause variations in the PCE of up to a few percent. In Fig. 2c, this effect can account for some experimental PCEs being higher than the simulated PCEs. In Fig. 2d and e, on the other hand, all but one of the experimentally determined PCEs surpass all simulation light source scenarios, despite additional losses being present in the real devices due to parasitic shunt and series resistances; *i.e.*, the real devices are expected to exhibit an experimental PCE lower than the thermodynamic limit. To explore this effect further, Fig. 2f shows the PCE measured experimentally (PCE_meas_ as filled symbols) at an irradiance *P*_source_ ≃ 0.3 mW cm^−2^ (or an illuminance around 1000 lux), which has been normalised to the corresponding PCE in the thermodynamic limit (PCE_therm_) under the different light sources. Each data point corresponds to an individual light source, the boxes indicate the interquartile range of the data, and the whiskers indicate the minimum and maximum. Intuitively, as System B and C possess more spectral variation in their above-gap EQE_PV_ (*cf.*Fig. 2b), then changes in spectral overlap between light sources produce larger variations in the simulated PCEs for these systems than for System A. This, of course, is why reference cells that exhibit a spectrally flat EQE_PV_ above the gap are desirable for calibrating PV testing apparatus.

By evaluating the difference between each system's minimum and maximum normalised PCE in Fig. 2f, it appears that changes in spectral overlap across the different 3000 K LEDs can, at most, account for a PCE comparison imprecision of up to 5%. In light of these findings, the indoor PV measurements can be categorised in one of two ways: (i) thermodynamically valid – where the experimentally determined measurements lie below or within the 5% spectral uncertainty for one or more of the light sources (*e.g.*, System A), giving PCE_meas_ ≤ PCE_therm_; or (ii) thermodynamically implausible – where PCE_meas_ > PCE_therm_ for all light sources (*e.g.,* System B and C), meaning that inconsistencies in the spectral irradiance of the light source alone cannot account for the mismatch between measured and simulated power outputs. As discussed shortly, it is possible that other sources of uncertainty can compound with the uncertainty in the light spectrum, which may increase the comparison imprecision.

### 2.3. Temporal instability of the light source

Retaining focus on the imprecision associated with the light source (for now), then some additional variation could be expected due to a temporally unstable output. Compared to the other experimental factors described in this work, retroactively quantifying the comparison imprecision associated with any temporal instability in the light source is infeasible as many publications do not report such data. However, recent findings by Parsons *et al.* suggest that an LED with a thermally stabilised output (after around 30 minutes of operation) will produce temporal instabilities on the order of 0.015%.^25^ However, these values may generally depend on the type of LED, its driving current, how aged it is, and whether or not is has thermally stabilised before being used to characterise an indoor PV device. In line with IEC TS 62607-7-2:2023, temporal instabilities of up to 2% are permissible for all but the poorest-rated light sources (which have temporal instabilities of up to 10%).^25,31^ Consequently, for this analysis temporal instability is assumed to account for up to 2% imprecision when comparing irradiance values. As this influences both the light power impinging on and short-circuit current produced by the DUT, then such imprecision propagates to a 2.8% imprecision in the PCE. As the irradiance on the DUT is usually calibrated using a reference cell, it has been assumed to represent an independent realisation of the temporal stability of the light source, such that the imprecisions are combined in quadrature.

### 2.4. Light attenuation

Following the choice and temporal stability of the light source used to characterise an indoor PV device, the next experimental factor shown in Fig. 1 that can inadvertently modify the lineshape of the spectral irradiance is the chosen attenuation technique. To explore this effect further, in this section the deviations in spectral irradiance and resulting uncertainty in device performance are considered for four different attenuation techniques. The most accurate of these techniques (whilst also being the most complex and costly to implement) are digital micromirror devices (DMDs).^25^ These arrays of micromirrors reflect the light emitted from a source onto the DUT. As each mirror of the DMD can be programmatically switched “on” (*i.e.*, reflecting light towards the DUT) or “off” (*i.e.*, reflecting away from the DUT) then the resulting irradiance on the DUT can be controlled to a high accuracy. If the mirrors have a spectrally flat reflectance across the visible region of the electromagnetic spectrum then minimal deviation will occur compared the spectral irradiance of the light source. This is demonstrated in Fig. 3a, where the spectral modification factor (*M*_at_) of a DMD was calculated as the ratio between the attenuated irradiance and the unattenuated irradiance. The effective irradiance incident on a DUT can be calculated by multiplying *M*_at_ with the unattenuated spectral irradiance of the light source (*I*_source_), and then re-scaling to the measured light intensity. Note that the image inset in Fig. 3a shows a typical DMD.^48^ As the DMD has a relatively flat *M*_at_ until the peripheries of the visible region of the electromagnetic spectrum, where LED emission spectra tend towards zero (*cf.*Fig. 2a), then DMDs make minimal modifications to the spectral irradiance of a light source.

**Fig. 3:**
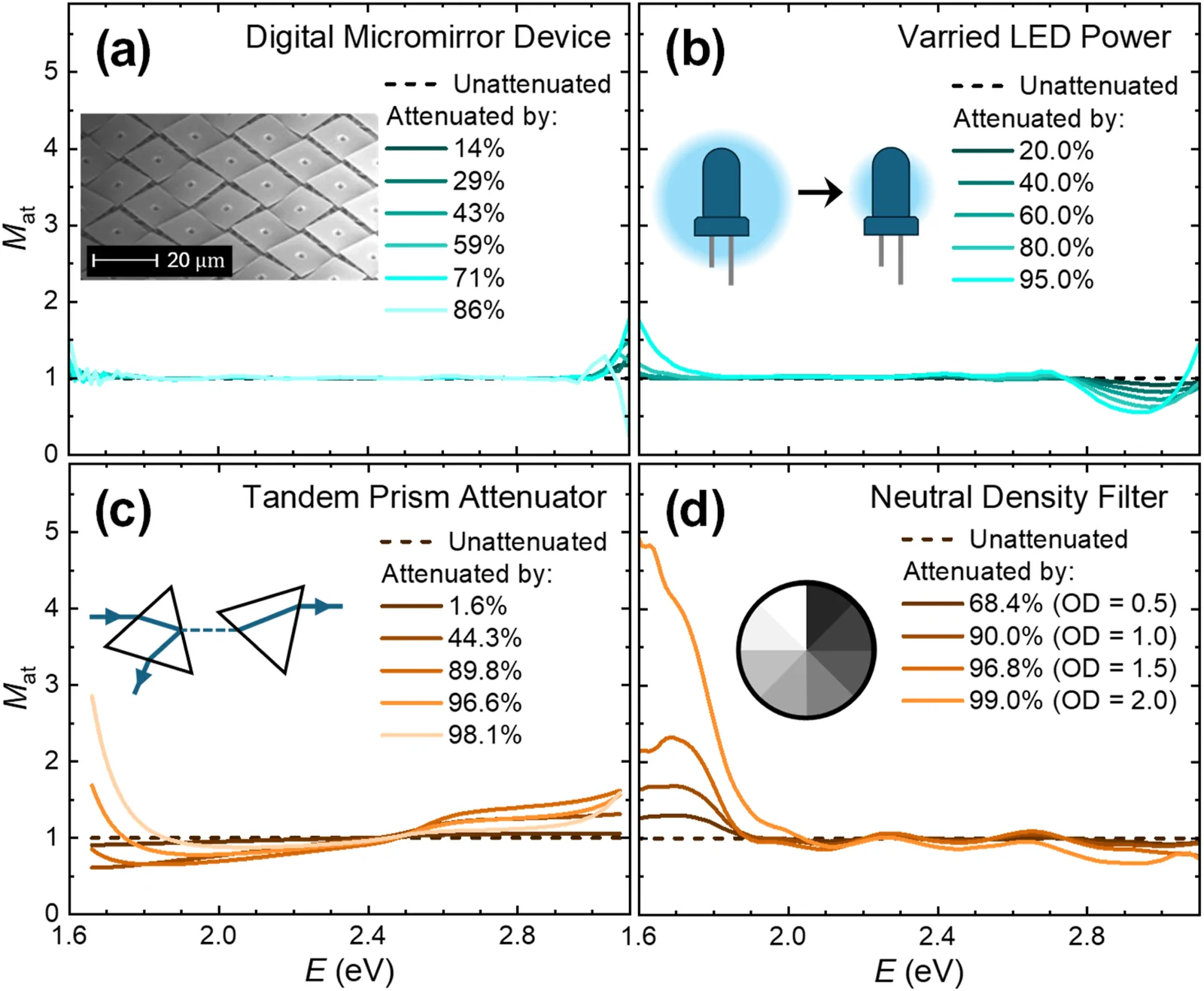
Spectral modification factor (*M*_at_) attributed to different light attenuation techniques, including (a) highly engineered digital micromirror devices (DMDs),^25^ (b) varied LED driving power,^28^ (c) tandem prism attenuators,^28^ and (d) neutral density filters.^28^ In each panel, two orders of magnitude of attenuation are considered, and an illustration of each technique has been inset in each panel. For (a), the inset scanning electron microscope image has been reproduced from ref. 48 with permission from Science Direct. For light attenuation by reduced LED power, a 4000 K LED was considered in the original work; *M*_at_ may vary from one type of LED to another.

A second technique for reducing the light intensity on a DUT is simply reducing the LED driving power, which – alongside reducing the number of emitted photons – may also reduce the temperature and alter the emission properties of the LED. As shown in Fig. 3b, the spectral modification resulting from reducing the driving power of a 4000 K LED primarily affects higher-energy photons, though this is largely unchanged across the rest of the visible region.^28^ The same cannot be said for the final two techniques considered in this section, which are prism attenuators and neutral density filters, the spectral modification factors of which are shown in Fig. 3c and d,^28^ respectively. Both techniques involve transmitting the light emitted by a source through a medium with some spectrally non-uniform transmittance (*T*); for neutral density filters, this transmittance relates to the optical density (OD) *via T*(*E*) = 10^−OD(*E*)^.^28^ As the variations in transmittance increase, the associated *M*_at_ leads to larger changes in spectral irradiance on the device. As a result, higher imprecisions could be expected when comparing the performance of indoor PVs characterised using such methods.

To determine the comparison imprecision introduced by the four attenuation techniques described above, the PCE was simulated in the thermodynamic limit for the three indoor PV systems considered in Fig. 2. For this investigation, the spectral modification factors of each attenuation technique were combined with the spectral irradiance of the 3000 K LED spectrum published by Wang *et al.* (which yielded the highest PCEs for all systems in Figure 2),^43^ before being renormalised to the required irradiance. As factors such as the distance from and driving power of the light source are often not readily available, two orders of magnitude were considered as a suitable range of attenuation to satisfy the 50 lux to 1000 lux values specified in IEC TS 62607-7-2:2023. The attenuated spectra were then used to simulate the thermodynamic-limit PCEs for each system, with the irradiance-dependent results being shown in SI Note 2. For each of the attenuation techniques, Fig. 4 shows the experimentally measured PCEs (PCE_meas_) of each of the three systems at an illuminance around 1000 lux normalised to attenuated PCEs simulated in the thermodynamic limit (PCE_therm_); similar to the process used for Fig. 2f.

**Fig. 4:**
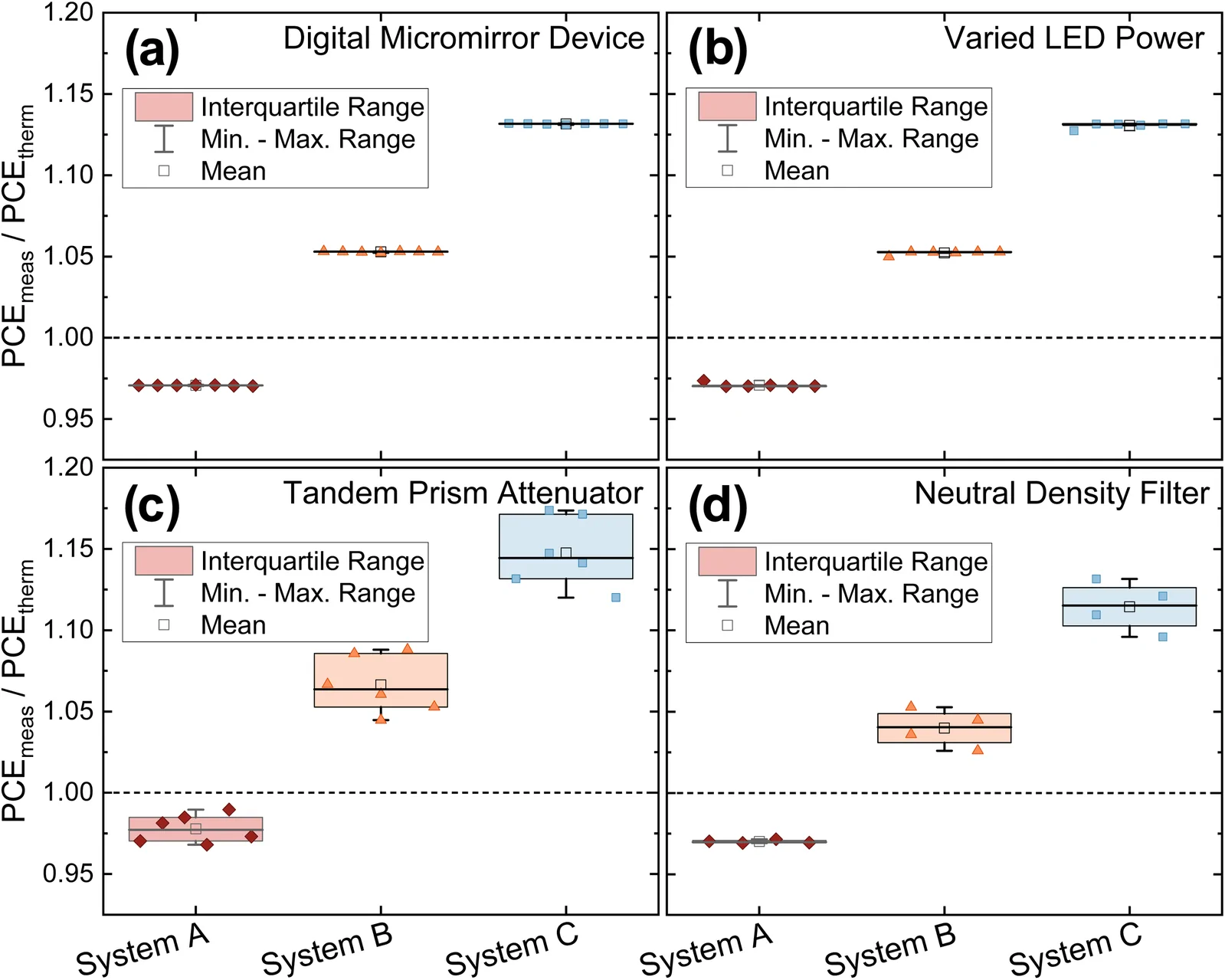
Evaluating comparison imprecision for the power conversion efficiencies (PCEs) of the three example systems considered in Fig. 2 (labelled as System A–C) for four different light attenuation techniques; namely (a) digital micromirror devices (DMDs), (b) varied LED driving powers, (c) prism attenuators, and (d) neutral density filters. For this analysis, the irradiance incident on the device-under-test was determined by multiplying each attenuation technique's spectral modification factor with the spectral irradiance of the 3000 K LED described by Wang *et al.*,^43^ which yielded the highest PCEs for each system in Fig. 2. For each of these systems, the resultant PCEs that were simulated in the thermodynamic limit (PCE_therm_) were then used to normalise the PCEs measured experimentally (PCE_meas_) at around 1000 lux, with the resultant spread in output data being shown by the box and whisker plots.

As expected, the DMD and varied LED power techniques lead to the smallest variation as the light is attenuated, as shown in Fig. 4a and b, respectively. Alternatively, the non-uniform spectral modification factors of the prism attenuator and neutral density filter techniques lead to attenuation-dependent PCEs, as shown in Fig. 4c and d, respectively. As with spectral variations between light sources from different manufacturers (*cf.* Section 2.2), devices with less spectrally uniform above-gap EQE_PV_ (such as System B and System C) undergo more changes in spectral overlap as the light is attenuated, leading to larger variation in the simulated PCEs. As a result, the spread in the data for Systems B and C is much broader than for System A, owing to the above-gap EQE_PV_ of the latter being more spectrally uniform. From Fig. 4, it's clear that the uncertainties arising from light attenuation closely relate to the chosen technique. Generously, the DMD or varied LED power techniques can account for 1% imprecision in PCE (at most). Prism attenuators and neutral density filters, on the other hand, can account for about 5% imprecision. Thus, while these latter techniques are perhaps easier to employ, they come with the trade-off of higher spectral modification and therefore higher imprecision.

### 2.5. Geometric spatial non-uniformity

With the comparison imprecision arising from variations in light source and through light attenuation now quantified, the next experimental factor we consider is a spatially non-uniform distribution in the irradiance across the full area of the DUT. Beyond the SNU inherent in the light source, SNU can be added *via* the geometry of the apparatus. For the discussion presented here we assume a perfect point source with no SNU at the source of emission, leading to a reasonable description for the imaging systems that are often used in solar simulators. As noted previously, the geometric SNU becomes prominent when the distance (*h*) between the DUT and the light source is too small relative to the side-length of the DUT (*w*). Assuming the irradiance on the surface of the DUT at a distance *r* from its centre can be described using the inverse-square law as *P*_source_(*r*) = *P*_source_(0) × *h*^2^/(*h*^2^ + *r*^2^), then by defining the SNU as the normalised difference between the irradiance at that distance and at its centre, it follows that^28^2
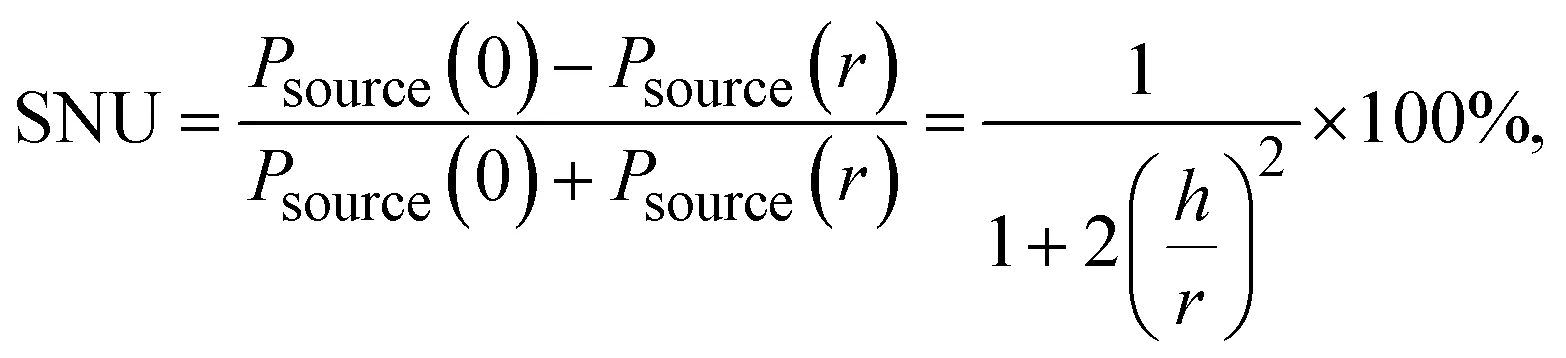


The maximum SNU occurs at the extremities, where 
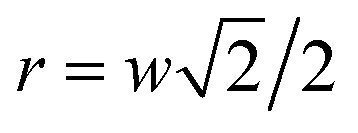
 and 
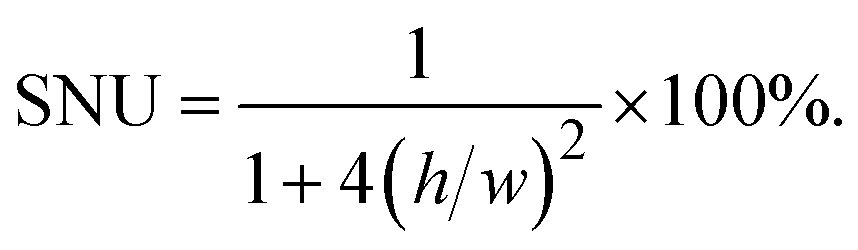
 If the device has a small enough area, or is sufficiently far from the light source, then *h* ≫ *r* and eqn (2) gives SNU → 0 across the whole device.

The effect of geometric SNU is explored in Fig. 5a–c for various ratios of *h*/*w*. In the case that *h* = 3*w*, which for a commercial-scale device with *w* ∼5 cm means that *h* = 15 cm, SNUs can reach 3% at the extremities. In other words, the irradiance at the centre of the device is 3% brighter than at the extremities. In the case that *h* = 10*w*, which is a ratio more akin to the testing of a small-area (*w* < 1 cm) laboratory device at the same height, the SNU drops to <0.25%. The effect of the *h* to *w* ratio on the maximum SNU is explored in more detail in SI Note 3. As details such as the distance from the light source to the DUT are often not readily available, we herein assume a generous SNU of 1% in the irradiance across laboratory-scale DUTs. As the irradiance feeds directly into the calculation of the PCE, through both the short-circuit current and the illumination power (which are measured independently), then this 1% uncertainty in irradiance combines in quadrature to yield a 1.5% PCE comparison imprecision.

**Fig. 5:**
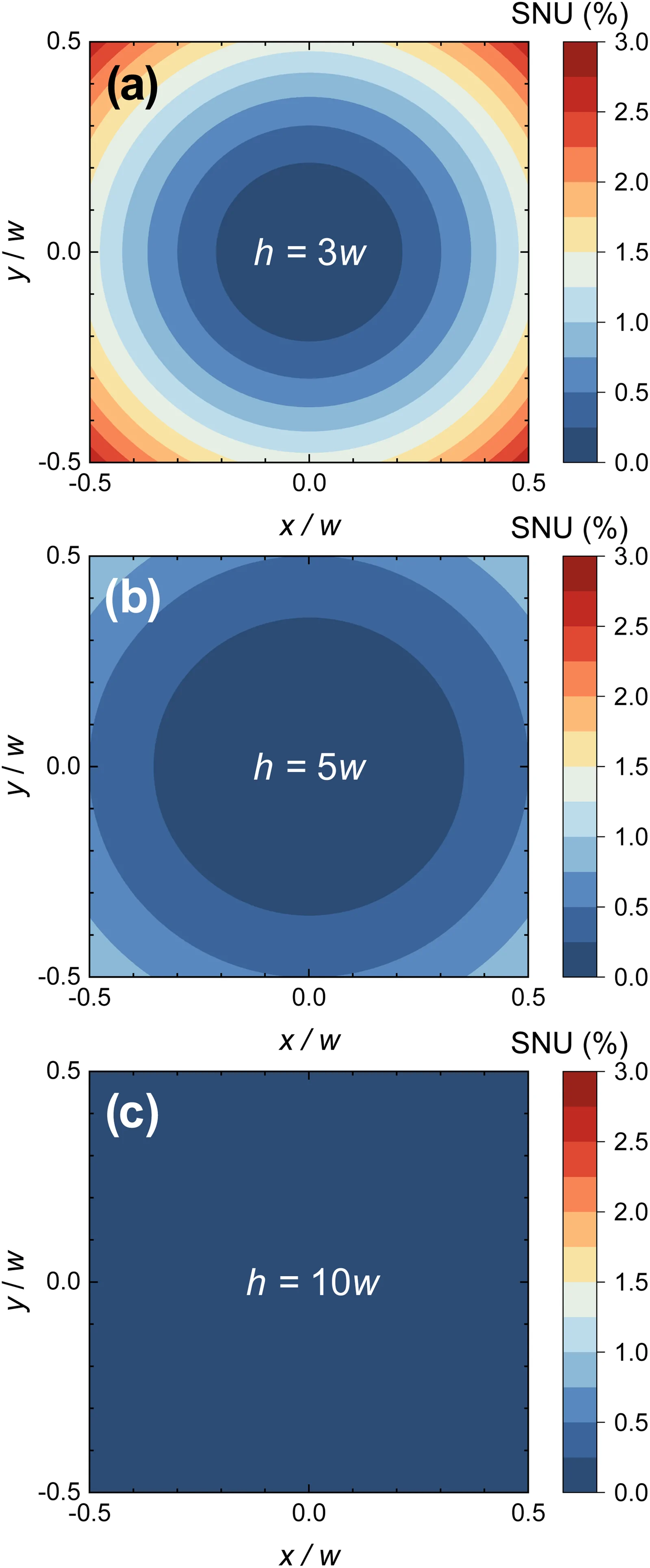
Spatial non-uniformity (SNU) in the irradiance across an indoor photovoltaic device-under-test (DUT) due to varying distance from a point source a distance *h* away. By varying the ratio of this distance to the width (*w*) of the device, the resultant SNUs calculated using eqn (2) are shown in (a)–(c), wherein *h* = 3*w*, *h* = 5*w*, and *h* = 10*w*, respectively.

### 2.6. Deviations in angular spectral response

In the preceding discussions, light was assumed to impinge on the device at normal incidence, with no additional reflectance losses due to Fresnel angular dependencies. However, further measurement uncertainties may be introduced in some experimental apparatus through the use of ‘pseudo-collimated’ light sources, which have some non-negligible angular distribution. Angular distributions can introduce uncertainties in the light intensity at the imaging plane as the angular response of the reference cell used to fix the light intensity may differ from the angular response of the DUT, leading to charge and current densities that vary from the case in which the two angular responses are identical (or if the light is collimated). To explore how such considerations affect the power output of indoor PV devices, a case study is presented in Fig. 6 using a custom-made silicon reference cell with a KJ4 optical filter (herein referred to as the reference cell), and an organic semiconductor-based indoor PV cell from Epishine (OC15-20x80-200; herein referred to as the OPV cell).

**Fig. 6:**
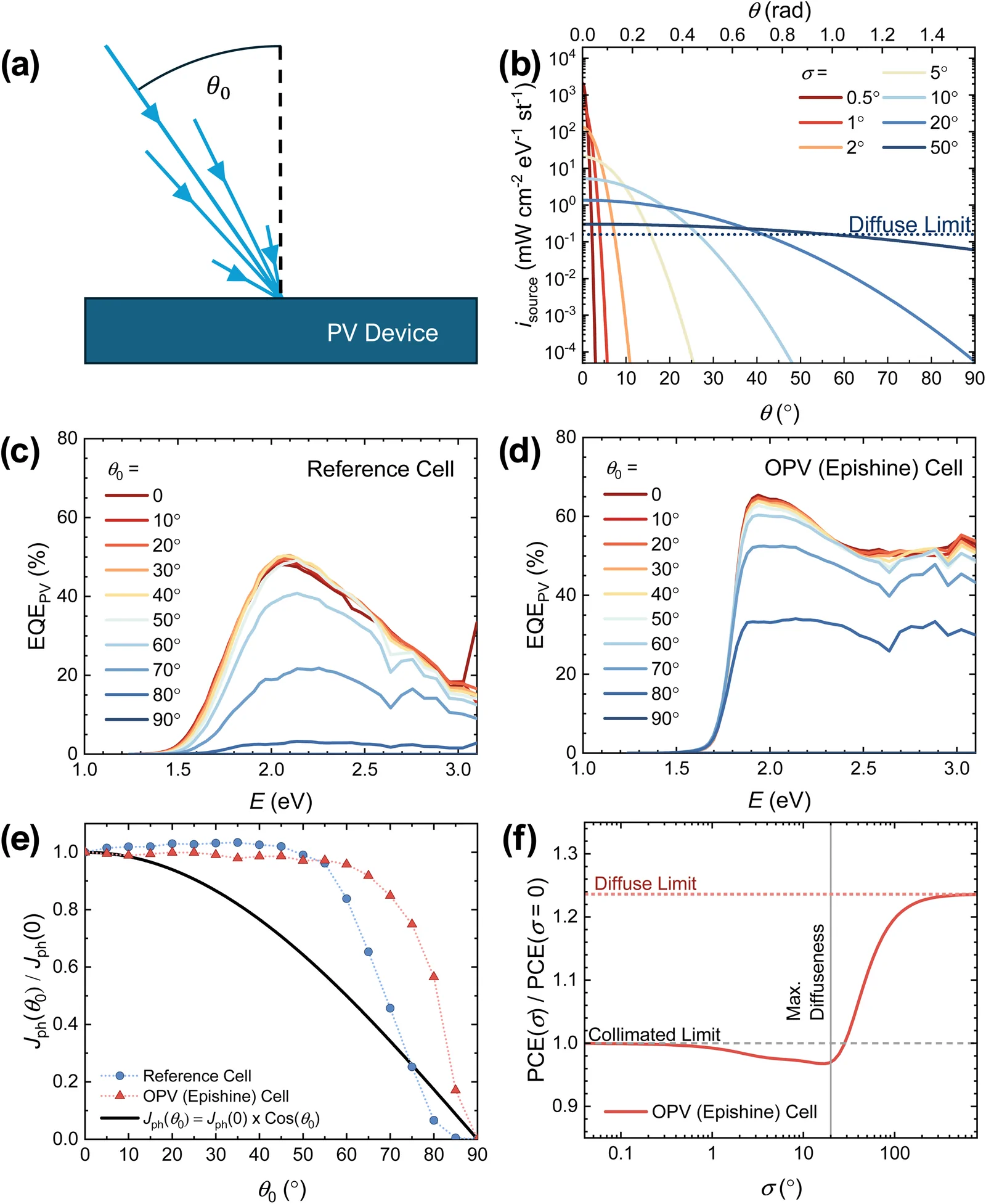
Calculating uncertainty due to differences between the angular spectral responses of a reference cell and a device-under-test. (a) Illustration of light impinging on a PV device obliquely, with a maximum occurring at angle *θ*_0_ relative to the normal. (b) Gaussian models for the angular distribution of incident light centred at *θ*_0_ = 0  using varied standard deviation (*σ*), which have been simulated using eqn (3) and (4); for derivation of these equations, see SI Note 4. The angular-dependent, photovoltaic external quantum efficiency (EQE_PV_) spectra measured for a custom-made, silicon-based reference cell and an organic photovoltaic (OPV) cell from Epishine are plotted *versus* photon energy (*E*) in (c) and (d), respectively. (e) Normalised angular-dependent photocurrent density (*J*_ph_) calculated using the EQE_PV_ spectra from panel (c) and (d) under CIE LED-B4 illumination. The solid black curve indicates a cosine approximation for the angular dependence of *J*_ph_. (f) Power conversion efficiency of the OPV (Epishine) cell under Gaussian-distributed CIE LED-B4 illumination of varied width (*σ*), where the light is centred at *θ*_0_ = 0. A target irradiance of at *P*_source_ = 0.3132 mW cm^−2^ was used; though for each *σ* value the irradiance was rescaled such that the reference cell produced the same photocurrent as obtained in the collimated case. The curve has been normalised to the value in the collimated limit (*σ* → 0), which is indicated by the dashed line. Whereas the dotted line indicates the output in the fully diffuse (*σ* → ∞) limit. The solid, vertical line roughly indicates the maximum tolerable level of diffuseness before the change in angular response drastically affects performance.

To begin, an illustration of pseudo-collimated light impinging on an indoor photovoltaic device at non-normal (oblique) incidence is provided in Fig. 6a, where most light makes an angle *θ*_0_ relative to the normal (dashed line), while the rest of the light has some distribution about this maximum. In this section, the angularly distributed spectral irradiance (*i*_source_) is described with a Gaussian distribution centred at *θ*_0_ = 0. After determining a suitable normalisation pre-factor to ensure that an integral of *i*_source_ over a hemispherical solid angle satisfies 
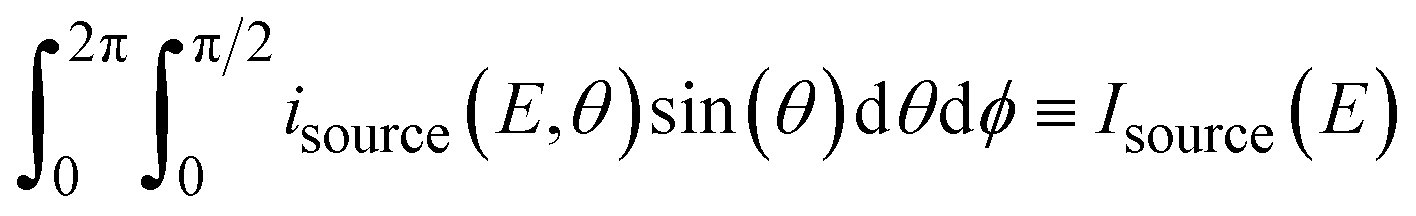
, this distribution can be written as (see SI Note 4)3
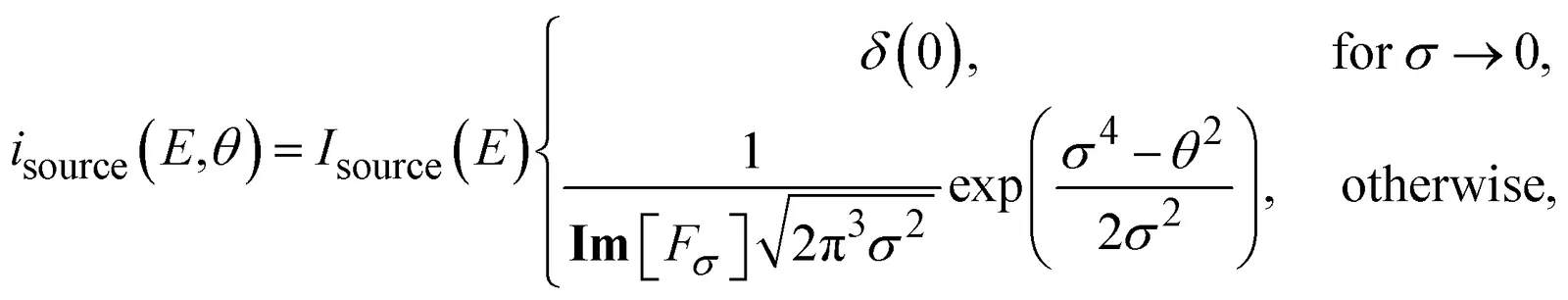
where *δ* denotes the Dirac delta function, while the complex quantity *F*_*σ*_ is defined by4
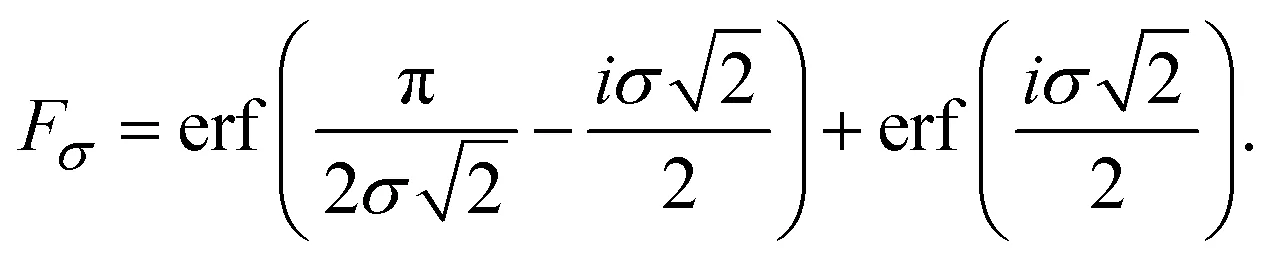
Here, 
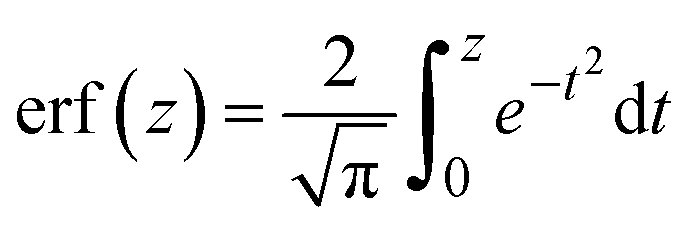
 is the error function. In eqn (3) and (4), the standard deviation of the Gaussian distribution (*σ*) has units of radians. Angularly distributed spectral irradiances simulated using these equations with varying *σ* (in units of degrees) are shown in Fig. 6b. As *σ* increases from zero, the angular spectral irradiance becomes distributed across a broader range of incident angles, with the peak value decreasing by orders of magnitude. For pseudo-collimated light with narrow distributions (0° ≤ *σ* < 5°), most light impinges near normal incidence, with the angular spectral irradiance decreasing by orders of magnitude (relative to the peak value) within the first 20°. The amount of light impinging near grazing incidence (*θ* → 90°) remains orders-of-magnitude smaller than the peak value until *σ* becomes very large. Ultimately, in the limit that *σ* → ∞ (representing completely diffuse light) the spectral irradiance becomes evenly distributed across all incident angles.

Both the angular dependence of light transmission and reflection at interfaces between electromagnetically distinct media in the device, as well as the path length of light propagation in each layer, will lead to an angular dependence of absorption and, hence, EQE_PV_.^49,50^ If a device's EQE_PV_ remains mostly invariant with respect to changes in incident angle, then its power output will be unaffected (as most of the light will still be converted into photocurrent). Alternatively, if its EQE_PV_ is highly sensitive to changes in incident angle, then this may not be the case. The experimentally determined, angular-dependent EQE_PV_ of the aforementioned reference cell and OPV cell are shown in Fig. 6c and d, respectively. A description of the experimental apparatus used to measure these angular-dependent EQE_PV_ spectra is provided in SI Note 5. For small angles, deviations in EQE_PV_ are minimal for both cells. Alternatively, for large angles EQE_PV_ begins to decrease for both cells as their reflectance increases. Note that these angular dependencies will closely relate to the refractive indices of the materials in the considered device, as well as the texture of the surfaces and interfaces. Using these angular-dependent EQE_PV_ spectra, the resultant integrated photocurrent densities (*J*_ph_) generated under CIE LED-B4 illumination were determined as a function of incident angle, with the normalised results plotted in Fig. 6e. In addition, a cosine approximation for the photocurrent density has been plotted as the solid line. From this figure, it's clear that at higher incident angles the reference cell exhibits a much greater angular dependence than the OPV cell, most likely due to an increased reflectance off the cover glass at its top. In any case, a prominent change in angular response indicates poor coupling of light into the device.

The inconsistency between the angular responses of a reference cell and a DUT manifests as an uncertainty in the relative responses of the devices under diffuse and collimated light. For example, measuring under perfectly collimated light would result in both the correct calibration of the light source and the correct measure of the DUT's PCE. However, for pseudo-collimated (or even diffuse) incident light the differing angular responses could result in a calibration that does not represent the response of the cell under the same light intensities. In other words, the charge density in the DUT under 1000-lux collimated or 1000-lux pseudo-collimated light may not be the same if the angular response of the DUT is not accounted for. For example, if calibrating to *P*_source_ = 0.3132 mW cm^−2^ (or 1000 lux under LED-B4), then as the light becomes more diffuse the reference cell's response will decrease, which in turn leads to a higher irradiance being required to achieve the expected response at that intensity. However, as the response of the DUT exhibits better light coupling (in this example), this increase in irradiance will manifest as an increase in charge density leading to an inadvertent increase in PCE, through the photocurrent, which would not have been otherwise present in the perfectly collimated case or in the case where the angular responses were identical.

To explore this effect further, the PCE of the OPV cell was simulated under increasingly diffuse LED-B4 illumination, where for each *σ* value the intensity was fixed using the reference cell's angular spectral response. A target light intensity *P*_source_ = 0.3132 mW cm^−2^ was used, under which the reference cell would produce a photocurrent of 54.92 µA cm^−2^ in the perfectly collimated case. However, in the non-collimated case, the total photocurrent generated by the reference cell was determined by the integrating the incident light across the hemispherical solid angle and then integrating over the whole spectrum using5
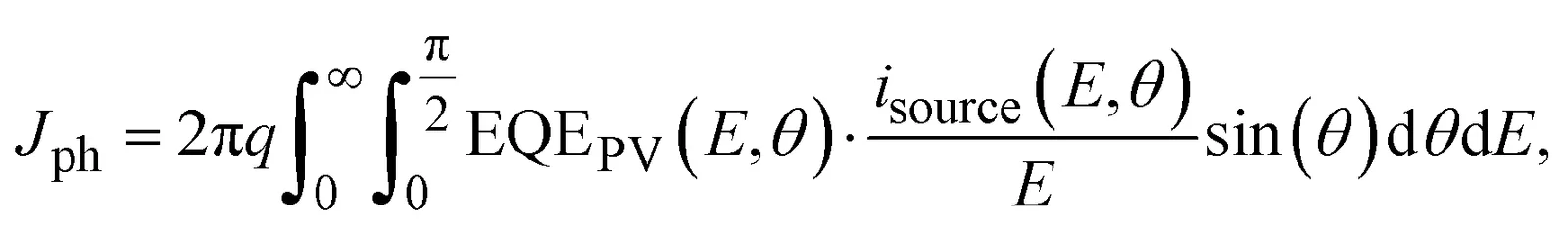
where *i*_source_(*E*,*θ*) is given by eqn (3). As the light becomes more diffuse, a higher irradiance is required to maintain the same *J*_ph_ in the reference cell as expected in the collimated case. Using this rescaled irradiance, the PCE of the OPV cell was evaluated in the thermodynamic limit using the procedure outlined in SI Note 1 – the results of this investigation have been normalised to the value in the collimated limit (where *σ* = 0) and then plotted *versus σ* in Fig. 6f. From this figure, it's clear that for pseudo-collimated light (small *σ*), the PCE of the OPV cell is almost indistinguishable from the perfectly collimated limit. As the light becomes more diffuse, however, variations between the angular spectral responses of the reference cell and the OPV cell result in the PCE of the latter decreasing by up to 3% relative to its collimated-limit value. To minimise imprecision when comparing reported data, the angular responses of reference cells and DUTs should ideally be closely matched. Alternatively, from a more practical perspective, the angular distribution of the light should be minimal.

Ultimately, beyond some maximum “diffusiveness” that the combination of devices considered in Fig. 6 can tolerate (which is at roughly *σ* = 20°), the reduction in angular response of the reference cell, coupled with the increased intensity and amount of light impinging at higher angles, leads to more power being generated in the OPV cell and an increase in the apparent PCE. This approaches the perfectly diffuse limit asymptotically as *σ* → ∞. In this limit, the PCE of the OPV cell is almost 25% higher than its value in the collimated limit. To minimise the comparison imprecision associated with differing angular spectral responses during indoor PV characterisation, collimated or pseudo-collimated (*i.e.*, sources with *σ* < 5°) light sources are preferable.

This, of course, is a highly system-specific problem; for the combination of reference cell and DUT considered in this section, differences in angular spectral response could at most account for up to 3% uncertainty in PCE (below the maximum diffuseness threshold). As the OPV cell is laminated with limited interfaces, the coupling of pseudo-collimated light into it is expected to be relatively efficient (as demonstrated by Fig. 6e), and an uncertainty of 3% is an expected lower limit on this value. Based on this, we therefore assign a minimum 3% imprecision to comparisons between indoor PV testing apparatus, where the light may be pseudo-collimated. It is important to note, however, that the imprecision determined for the combination of devices considered in this work is not being trivially applied to all other combinations of devices, as the angular responses of reference cells and DUTs are highly architecture- and material-dependent; instead, the 3% imprecision assigned here is an illustrative allowance based on one combination of devices. For any specific system the uncertainty could be much greater, particularly as the light becomes more diffuse. Importantly, this effect will not necessarily reduce the measured PCE, with Fig. 6f demonstrating an increase in PCE for highly diffuse light, which arises from the highly efficient coupling of high-angle light of the OPV cell. If, for instance, the DUT exhibited poorer light coupling than the reference cell the PCE would decrease in the perfectly diffuse limit. Again, such observations may change depending on the combination of reference cell and DUT, as it is exceedingly unlikely that they will have identical angular responses and thus respond the same way to collimated and uncollimated light.

### 2.7. Combining sources of uncertainty and imprecision

With several different experimental factors that lead to comparison imprecision now audited, these uncorrelated sources can be combined to yield a total imprecision associated with comparing the PCEs of indoor PVs from different publications. As a reminder, the estimated upper bounds on the individual imprecisions assigned to each source are as follows: (i) for a given light source CCT, the imprecision in PCE due to spectral variation between light sources from different manufacturers could account for up to 5% discrepancy (despite all sources holding the same CCT); (ii) the temporal instability in all but the poorest-rated light sources can account for up to 2.8% PCE comparison imprecision; (iii) technique-dependent light attenuation can account for at most 5% imprecision (being less than 1% for DMDs and varied LED power but up to 5% for tandem prism attenuators and neutral density filters); (iv) SNUs produce a 1.5% imprecision in PCE; and (v) differences between the angular spectral responses of the reference cell used to fix the light intensity and the DUT produce a minimum 3% imprecision in PCE for pseudo-collimated light, though this imprecision shrinks if the light is more collimated, ultimately tending to zero in the perfectly collimated limit. We emphasise that the experimental factors that lead to comparison imprecisions are general to all indoor PV testing apparatus but specific to none. Because of this, we have considered a large upper bound to these imprecisions by assuming that the measurements themselves are precise and repeatable. In doing so, we highlight the experimental factors that lead to difficulties in comparing reported measurements and give the greatest chance of validating previously reported measurements.

As the upper bounds on the imprecisions identified above are uncertainty components estimated through physical models, rather than through repeat measurements, then according to the Joint Committee for Guides in Metrology (JCGM) they should be classified as Type B uncertainties. Assuming these imprecisions hold rectangular (uniform) uncertainty distributions, then the corresponding standard uncertainties can be determined by dividing each of them by 
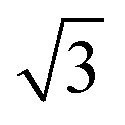
.^51^ This yields the following standard uncertainties: spectral variation (2.89%), temporal instability (1.62%), attenuation technique (2.89%), SNUs (0.87%), and differences in angular spectral response (1.73%). A summary of the estimated comparison imprecisions and standard PCE uncertainties due to the different experimental factors uncertainty is provided in Table 1. These standard uncertainties can be combined in quadrature to yield the total one-standard-deviation uncertainty associated with retrospective comparisons in the measured PCE,^52^ which is 4.80%. An expanded uncertainty interval can be obtained by multiplying this standard uncertainty with a coverage factor, which often lies in the range of 2 to 3.^51^ Taking a coverage factor of 2 expands the uncertainty interval to 9.60%, and the true PCE is expected to sit within this interval range with approximately 95% confidence. However, it is statistically possible that all sources of imprecision take their maximum value and act in the same direction, *e.g.*, spectral variations due to light source and attenuation technique can act in parallel with variations due to angular spectral response and temporal instability. Thus, a conservative, worst-case validation bound can therefore be created by adding the individual comparison imprecisions, with the maximum plausible cumulative deviation being 17.30%.

**Table 1:** A tabulated summary of the experimental factors considered in Sections 2.2–2.6 and their estimated contributions to the comparison imprecision and standard uncertainty in the power conversion efficiency (PCE)

Section	Experimental factor	Contribution to PCE comparison imprecision (%)	Corresponding standard uncertainty in PCE (%)
2.2	Spectral variation between light sources (with same CCT)	5.00	2.89
2.3	Temporal instability	2.80	1.62
2.4	Light attenuation	5.00	2.89
2.5	Geometric spatial non-uniformity	1.50	0.87
2.6	Deviations in angular spectral response	3.00	1.73

With these various uncertainties now clarified, it is possible that when attempting to validate the reported performance of indoor PVs using thermodynamic arguments, some systems that at first glance appear “thermodynamically implausible” might – due to the inherent uncertainty of the experiment itself or the imprecision in comparing unstandardised measurements – be plausible. Hence, in addition to the “thermodynamically valid” and “thermodynamically implausible” classifications introduced in Section 2.2, a third classification is now introduced: “thermodynamically plausible”, where the reported PCEs are not thermodynamically valid, but still lie with the 17.30% conservative, worst-case validation bound of the indoor PV measurement.

## 3. Validating state-of-the-art indoor photovoltaic measurements

In this section, our open-source computational tool, *PV-Simulator*,^36^ is used to analyse the thermodynamic limits of previously reported PV systems,and validate measurements using the total imprecision of Section 2.7. Fig. 7 shows the results of this analysis for several inorganic (grey squares), perovskite (purple circles), and organic (blue triangle) semiconductor-based indoor PVs. The reported PCEs measured experimentally under indoor testing conditions (PCE_meas_) were once again compared with the PCEs evaluated in the thermodynamic limit (PCE_therm_) using each system's EQE_PV_ spectrum and 
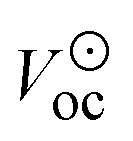
. As the indoor PVs were all measured under various light sources and intensities, the closest-available-CCT spectrum to the one reported in the measurement was used to evaluate PCE_therm_. As previously discussed in Section 2.2, an indoor PV may perform better under differing sources with identical CCT. Therefore, in the cases where multiple sources were available with the listed CCT (3000 K LED, for example) the source resulting in the largest PCE_therm_ was utilised. Note again that parasitic resistances were assumed negligible in these thermodynamic-limit calculations, though these are undoubtedly present in the real devices and will reduce PCE_therm_, pushing more systems from being rated thermodynamically valid to thermodynamically plausible, and from thermodynamically plausible to thermodynamically implausible.

**Fig. 7:**
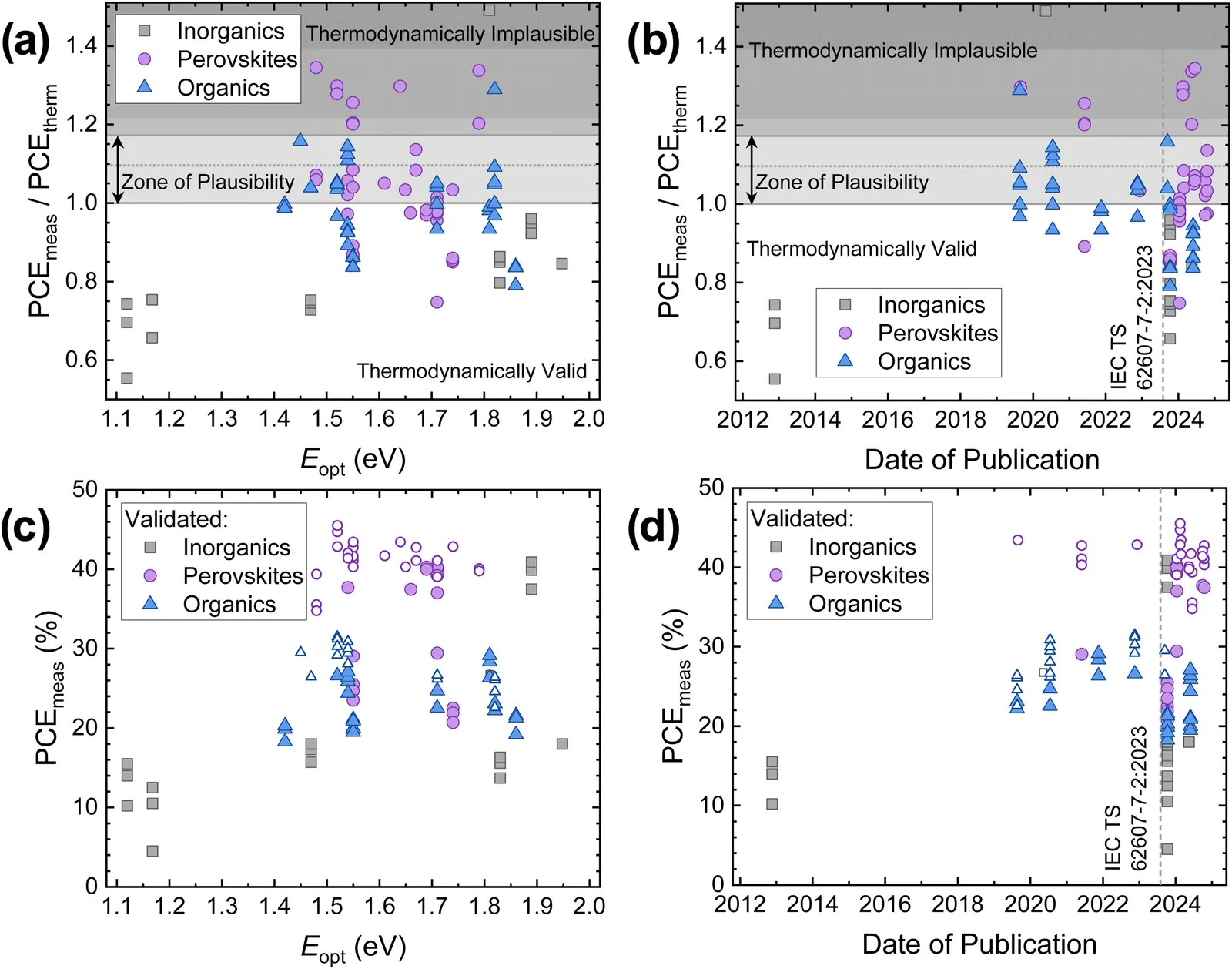
Validating reported performance of indoor photovoltaics based on inorganic semiconductors (grey squares), perovskite semiconductors (purple circles), and organic semiconductors (blue triangles) using thermodynamic, first-principles analysis. For each system, the ratio of the PCE measured experimentally (PCE_meas_) to the PCE determined in the thermodynamic limit (PCE_therm_) using its EQE_PV_ spectrum and 
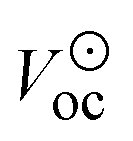
 is plotted *versus* (a) optical gap (*E*_opt_) and (b) publication date. In these panels, the dark grey shaded regions indicate where PCE_meas_ exceeds PCE_therm_, and so the measurements are classed as thermodynamically implausible. The lighter-grey region indicates a ‘zone of plausibility’, as defined by the conservative 17.30% upper bound for plausibility, in which all comparison imprecisions are at maximum and act in tandem. The dotted horizontal lines indicate the 9.60% expanded uncertainty interval calculated in Section 2.7, with the true normalised PCEs lying below this value with approximately 95% confidence. The thermodynamically valid PCEs are plotted as solid symbols against *E*_opt_ and publication date in (c) and (d), respectively, with the hollow symbols indicating measurements that either lie in the zone of plausibility or are thermodynamically implausible. Note that all measurements were made at light intensities ranging from 50 to 1000 lux. In (b) and (d), the vertical dashed lines indicate when indoor photovoltaic testing standard IEC TS 62607-7-2:2023 was introduced.^31^

From Fig. 7a and b, it appears that upwards of 50% of reported measurements of indoor PVs could be thermodynamically implausible, though many of these lie within the 17.30% conservative upper bound for plausibility described in the previous section – which is indicated by the so-called ‘zone of plausibility’ in Fig. 7a and b. In these panels, the dotted lines indicate the 9.60% expanded uncertainty also described in the previous section, with the true normalised PCEs lying within this range with approximately 95% confidence. From Fig. 7b, it is clear that even measurements reported in the last three years (after the introduction of indoor PV testing standard IEC TS 62607-7-2:2023) can be affected by these sources of uncertainty and imprecision.^31^ By considering systems with PCE_meas_ ≤ PCE_therm_, state-of-the-art, thermodynamically validated indoor photovoltaic measurements could be summarised and compared, as shown in Fig. 7c and d. Here, the filled-in data points indicate measurements that are validated, while the hollow data points either lie in the zone of plausibility, or are thermodynamically implausible. Once again, we emphasise the fact that further investigation on a more diverse range of experimental apparatus could broaden the zone of plausibility, thereby altering which measurements are classified as plausible and which are implausible (see SI Note 7). Nevertheless, the larger PCE_meas_ is relative to PCE_therm_, the more implausible it is likely to be.

From Fig. 7, we can see that the maximum validated PCEs of the perovskite, organic, and inorganic semiconductor-based indoor PVs considered in this work are 40.3%, 29.2%, and 40.9%, respectively. Note that all these PVs were measured at an irradiance *P*_source_ ≈ 0.3 mW cm^−2^ (or an illuminance around 1000 lux). Some reports of PCEs as high as 46% are thermodynamically implausible, even after accounting for conservative upper bounds on comparison imprecision. Summaries of the thermodynamically validated measurements for perovskite, organic, and inorganic semiconductor-based indoor PVs are provided in the SI as Tables S1–S3, respectively.

We note again that additional losses due to parasitic resistances were not accounted for in these calculations. In particular, the shunt (parallel) resistance can limit the performance of indoor photovoltaics under low light intensities, further lowering PCE_therm_. Consequently, some of the systems that have been classified as “thermodynamically valid” using the parasitic-resistance-free calculations may require reclassification following further scrutiny. Finally, we note that other factors can lead to small discrepancies between the experimental measurements and the thermodynamic-limit performance simulated using EQE_PV_ spectra. These factors include a manufacturer-specified photodiode calibration uncertainty associated with the EQE_PV_ measurement (typically around 1%; see SI Note 8), degradation of devices between measurements, and errors during EQE_PV_ digitisation (which was required for many systems as the raw EQE_PV_ data was not publicly available). Such discrepancies between measured and predicted performance are widely observed,^53^ and would typically result in an underestimation of PCE_therm_, resulting in more “validated” being systems pushed into the zone of plausibility.^54^

## 4. Conclusion

In recent years the number of publications reporting the power output of indoor PVs under sources of artificial light has grown rapidly. However, many publications have not adhered to any set of standardised testing protocols, with the light sources, attenuation techniques, and levels of light collimation varying from one publication to another. Furthermore, many reports neglect to assign uncertainties due to spatial non-uniformities and temporal instabilities in the light. The effects of these different experimental factors were explored in this work, with the imprecision associated with each when comparing reported measurements being audited and combined. Ultimately, it was found that indoor PV device characterisation using non-standardised apparatus can account for up to 17.30% imprecision when comparing to other works. Additionally, the inherent uncertainty when measuring reference cells and devices under diffuse or pseudo-collimated light sources was described and, in an archetypal example, found to account for a minimum of 3% uncertainty in PCE under pseudo-collimated light. This uncertainty is tied to any differences between the angular responses of the reference cell used to fix the light intensity and the PV device-under-test; it drops to zero in the limit that the light is perfectly collimated.

The thermodynamic limit of previously reported indoor PVs was calculated under a variety of light sources using their respective EQE_PV_ spectra and non-radiative-open-circuit voltage losses. By comparing the thermodynamic limit with experimental reports, data measured with unstandardised apparatus were classified as either thermodynamically valid, plausible, or implausible. The thermodynamically validated performance of many state-of-the-art indoor PVs was summarised, and it was found that the maximum validated PCEs of the perovskite, organic, and inorganic semiconductor-based systems considered in this work were 40.3%, 29.2%, and 40.9%, respectively (at an irradiance *P*_source_ ≃ 0.3 mW cm^−2^, or an illuminance of around 1000 lux). This analysis highlights the requirement for both an international,standardised testing protocol and journal-level reporting metrics, such that comparison imprecisions between the reported PCEs of indoor PVs can be eliminated. The wide-spread adoption of a standardised testing protocol, even an imperfect one, will allow for robust comparisons between reported literature values, increase confidence in emerging technologies, and generate assurance for an industrial base to develop around indoor PV technologies.

To support reliable comparisons of unstandardised measurements and analyses more broadly, measurements and data for many indoor PV devices have been stored in our open-source and freely available computational tool, *PV-Simulator*.^36^ Moreover, this tool has been upgraded with capabilities for validating measurements of indoor PVs.

## Author contributions

AMK: conceptualisation; methodology; software; validation; formal analysis; investigation; resources; data curation; writing – original draft; writing – review & editing; visualisation. DBR: conceptualisation; methodology; investigation; data curation; writing – review & editing; supervision. GB: conceptualisation; methodology; writing – review & editing; supervision; project administration. PM: conceptualisation; methodology; writing – review & editing; supervision; project administration; funding acquisition.

## Conflicts of interest

The authors declare no conflicts of interest.

## Supplementary Material

EL-OLF-D6EL00150E-s001

## Data Availability

The data that support the results and conclusions of this work (including the EQE_PV_ spectra of all considered devices) are available online in the *PV-Simulator* repository on GitHub.^36^ The data used to generate the figures are likewise available in another GitHub repository.^55^ Finally, references to indoor photovoltaic systems that were found to not be thermodynamically valid were purposefully omitted to preserve the anonymity of the authors of those works. However, these references will be made available upon request from the Corresponding Authors. Supplementary information (SI): a description of the thermodynamic device model; additional irradiance and attenuation-dependent curves for the three considered systems; a discussion on the maximum spatial non-uniformity for different height-width ratios; additional angular-dependent EQE_PV_ measurements; a summary of how the width of the zone of plausiblity influences the share of measurements which are plausible; and tabulated summaries of the validated indoor PV measurements. See DOI: https://doi.org/10.1039/d6el00150e.
