# Supplementary material for: Validating and comparing reported indoor photovoltaic performance
Source: EES Solar. 2026 Oct 27. Online ahead of print. doi: 10.1039/d6el00150e (PMC13732177; doi:10.1039/d6el00150e)
Supplement: Supplementary file 1 [file EL-OLF-D6EL00150E-s001.pdf]

Supplementary Information (SI) for EES Solar.
This journal is © The Royal Society of Chemistry

Validating and Comparing Reported Indoor Photovoltaic Performance

Austin M. Kay[*1], *Gregory Burwell*[1], *Drew B. Riley*[1], *& Paul Meredith*[*1]

[1]Centre for Integrative Semiconductor Materials (CISM) and Department of Physics, Swansea University Bay Campus, Swansea, SA1 8EN, United Kingdom

Email: A.M.Kay@Swansea.ac.uk; Paul.Meredith@Swansea.ac.uk

Contents

Supporting Note 1: Simulating Indoor Photovoltaics 2
Supporting Note 2: Attenuation Technique-Dependent Performance 4
Supporting Note 3: Maximum Spatial Non-Uniformity 7
Supporting Note 4: Parameterising Diffuse Light 8
Supporting Note 5: Angular-Dependent EQE_{PV} Measurements 10
Supporting Note 6: Validated Literature Data (in Tabulated Form) 12
Supporting Note 7: Effect of Width of Zone of Plausibility 15
Supporting Note 8: Calibration Uncertainty for EQE_{PV} Measurements 16

Supporting Note 1: Simulating Indoor Photovoltaics

To simulate power generation in photovoltaic devices illuminated by sources of artificial light, a technique recently described by the authors was employed.[1] This technique combines a device's photovoltaic external quantum efficiency ($\mathrm{EQE_{PV}}$) spectrum with the spectral photon flux (Φ_{source}) of the considered light source to determine the photocurrent density (J_{ph}), dark saturation current density (J_0), open-circuit voltage (V_{oc}), and ultimately the maximum power point parameters; all while accounting for band-filling effects,[2] the chemical potential of radiation,[3] non-radiative open-circuit voltage losses, and the failure of the reciprocity principle for states that lie well below the gap.[4]

To begin, the photocurrent generated in a photovoltaic device under voltage bias V can be evaluated using its $\mathrm{EQE_{PV}}$ spectrum and the spectral flux of the light source via the general expression[5]

$$J_{\mathrm{ph}}(V) = q\int_{E_{\mathrm{lower}}}^{\infty} \mathrm{EQE_{PV}}(E)\cdot\tanh\left(\frac{E-qV}{4k_{\mathrm{B}}T}\right)\cdot\Phi_{\mathrm{source}}(E)\,\mathrm{d}E. \qquad \textbf{(S1.1)}$$

Here, E denotes the photon energy, while q is the elementary charge, k_{B} is Boltzmann's constant, and T is the temperature of the photovoltaic device. Note the lower limit of the integral (E_{lower}) accounts for deep trap states (with energies much smaller than the bandgap energy) not obeying the standard reciprocity relation,[4] while the hyperbolic factor accounts for the effects of (sub-gap) state-filling.[2] Generally, the largest contribution to the photocurrent is made by above-gap states with energy $E > E_{\mathrm{g}}$ (with E_{g} being the bandgap energy), and the hyperbolic correction factor tends to one for these states as $qV < E_{\mathrm{g}} < E$ at typical operating voltages for photovoltaic devices.[1]

The dark saturation current density can be determined using the spectral photon flux of ambient radiation inside the device (Φ_{Planck}), which may be written in terms of the device's temperature as

$$\Phi_{\mathrm{Planck}}(E) = \frac{2\pi}{h^3c^2}\frac{E^2}{\exp\left(\frac{E}{k_{\mathrm{B}}T}\right)-1}, \qquad \textbf{(S1.2)}$$

where h denotes Planck's constant and c is the speed of light in vacuum. Using this expression for ambient radiation, the dark saturation current density can be evaluated in the radiative limit ($J_0 \rightarrow J_0^{\mathrm{rad}}$) using

$$J_0^{\mathrm{rad}}(V) = q\int_{E_{\mathrm{lower}}}^{\infty} \mathrm{EQE_{PV}}(E)\cdot w(E,V)\cdot\Phi_{\mathrm{Planck}}(E)\,\mathrm{d}E. \qquad \textbf{(S1.3)}$$

In this case, the term $w(E,V)$ is a correction factor that accounts for state-filling effects and the chemical potential radiation;[3] it is defined by[1]

$$w(E,V) = \frac{1}{\left[1 + \exp\left(\frac{qV - E}{2k_\mathrm{B}T}\right)\right]^2}. \quad \textbf{(S1.4)}$$

For states that are well above the gap ($E \gg E_\mathrm{g} > qV$), it follows that $w(E,V) \to 1$. In practice, non-radiative open-circuit voltage losses ($\Delta V_\mathrm{oc}^\mathrm{nr}$) in the device increase the dark saturation current density, and so in the non-radiative limit, J_0 may be calculated from J_0^rad using

$$J_0(V) = \frac{J_0^\mathrm{rad}(V)}{\mathrm{EQE_{EL}}} = J_0^\mathrm{rad}(V) \exp\left(\frac{q\Delta V_\mathrm{oc}^\mathrm{nr}}{k_\mathrm{B}T}\right) \quad \textbf{(S1.5)}$$

where $\mathrm{EQE_{EL}} = \exp\left(-\frac{q\Delta V_\mathrm{oc}^\mathrm{nr}}{k_\mathrm{B}T}\right)$ is the device's electroluminescent external quantum efficiency.[6] In the radiative limit, non-radiative open-circuit voltage losses are negligible ($V_\mathrm{oc}^\mathrm{nr} \to 0$), giving $\mathrm{EQE_{EL}} \to 1$ and $J_0(V) \to J_0^\mathrm{rad}(V)$.

Using Equation **(S1.1)-(S1.5)**, the total current density (J) generated by a photovoltaic device under voltage bias V can be described using the ideal diode equation

$$J(V) = J_0(V)\left[\exp\left(\frac{qV}{k_\mathrm{B}T}\right) - 1\right] - J_\mathrm{ph}(V). \quad \textbf{(S1.6)}$$

By numerically evaluating the photocurrent, the dark saturation current, and the total current produced by the device under different voltage biases, Equation **(S1.6)** can be used to identify the maximum power point voltage (V_mpp), the corresponding current density (J_mpp), and the power conversion efficiency (PCE); which are defined by

$$\left.\frac{\mathrm{d}\,[J(V)\cdot V]}{\mathrm{d}V}\right|_{V=V_\mathrm{mpp}} \equiv 0, \quad \textbf{(S1.7a)}$$

$$J_\mathrm{mpp} \equiv J(V_\mathrm{mpp}), \quad \textbf{(S1.7b)}$$

$$\mathrm{PCE} \equiv \frac{J_\mathrm{mpp}\cdot V_\mathrm{mpp}}{P_\mathrm{source}}. \quad \textbf{(S1.7c)}$$

Here, $P_\mathrm{source} = \int_0^\infty E \cdot \Phi_\mathrm{source}(E)\,\mathrm{d}E$ denotes the integrated irradiance of the light source.

The approach described above was employed to simulate the power generated by the different material systems considered in each figure of the main text. To easily simulate power generation under different light sources, a freely-available computational tool, *PV-Simulator*,[7] has been constructed by the authors.[1] This tool currently holds the $\mathrm{EQE_{PV}}$ spectra of a few hundred photovoltaic devices from different material classes, as well as more than one hundred spectrally-unique light sources; enabling a quick comparison of indoor photovoltaic performance under a variety of illumination conditions.

Supporting Note 2: Attenuation Technique-Dependent Performance

In **Section 2.4** of the main text, the uncertainty induced by different light attenuation techniques was quantified for three systems (labelled as Systems A-C), where the PCE was simulated for each system in the thermodynamic limit using the methodology described in **Supporting Note 1**. For this investigation, a range of effective light sources were considered. These sources were built from a 3000K LED spectrum described by Wang et al.[8] by multiplying it with the spectral modification factors attributed to the different levels of attenuation of the different techniques. These spectra were then rescaled to the desired irradiances. The results of this analysis are shown for System A, System B, and System C in **Figure S1**, **Figure S2**, and **Figure S3**, respectively. The data points nearest to an illuminance of 1000 lux (or an irradiance $P_{\text{source}} = 0.3132\ \text{mW cm}^{-2}$) were considered in **Figure 4** of the main text.

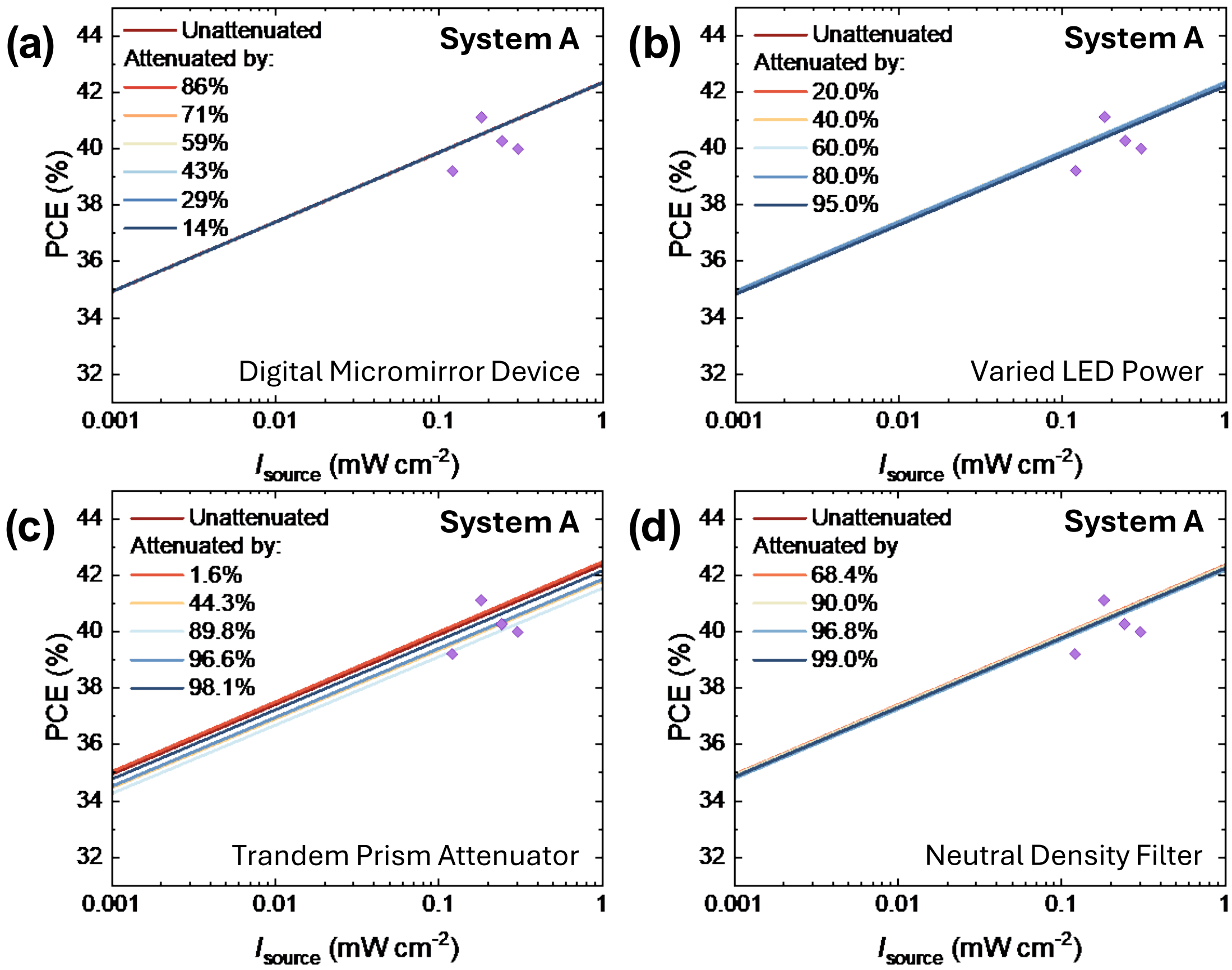

Figure S1: Power conversion efficiency (PCE) simulated using the photovoltaic external quantum efficiency spectrum and non-radiative open-circuit voltage loss of System A then plotted versus irradiance for four different attenuation techniques; namely these techniques are **(a)** a digital micromirror device, **(b)** varied LED driving powers, **(c)** a tandem prism attenuator, and **(d)** a neutral density filter. In all panels, the purple diamonds indicate the experimentally-determined measurements.

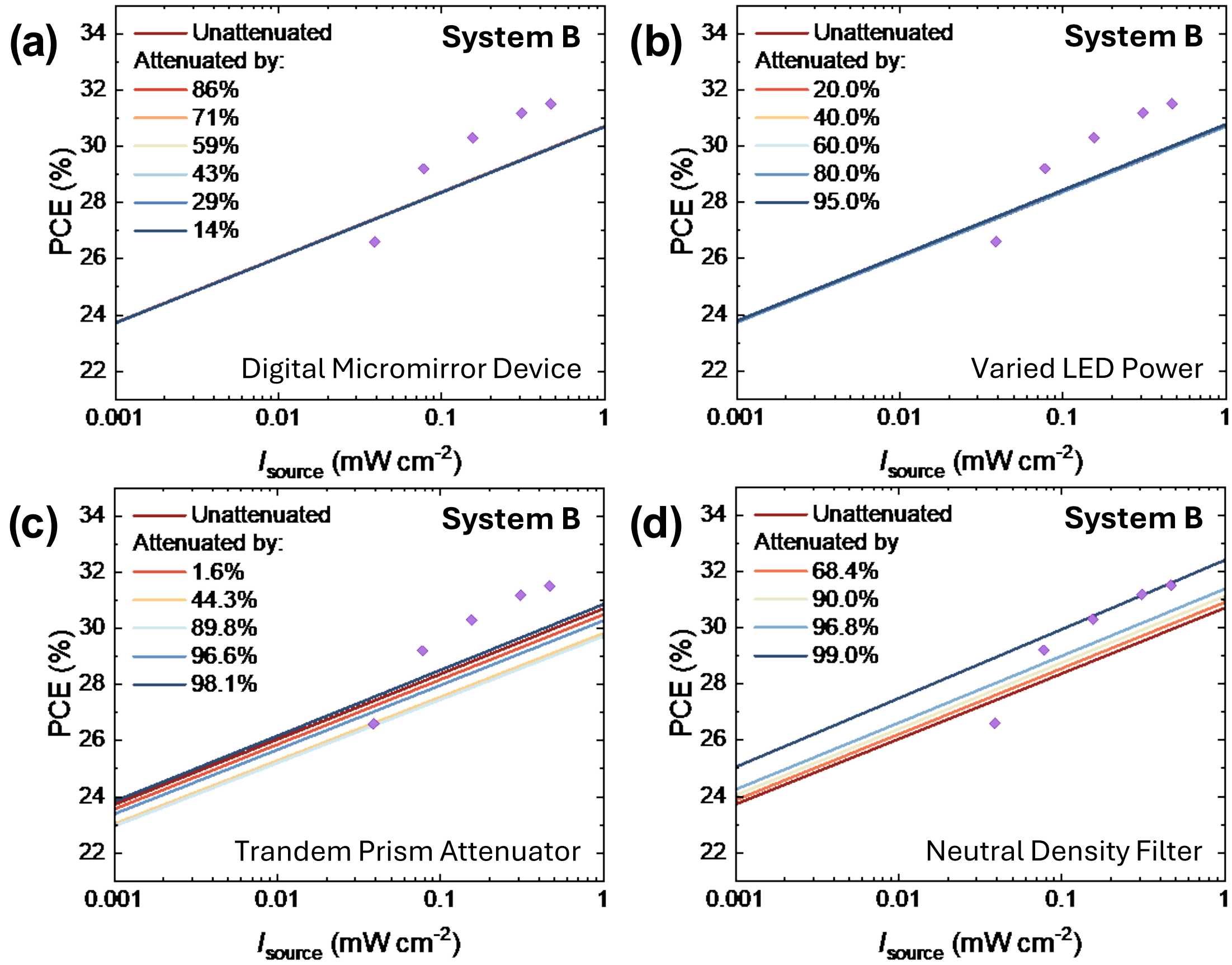

Figure S2: Power conversion efficiency (PCE) simulated using the photovoltaic external quantum efficiency spectrum and non-radiative open-circuit voltage loss of System B then plotted versus irradiance for four different attenuation techniques; namely these techniques are **(a)** a digital micromirror device, **(b)** varied LED driving powers, **(c)** a tandem prism attenuator, and **(d)** a neutral density filter. In all panels, the purple diamonds indicate the experimentally-determined measurements.

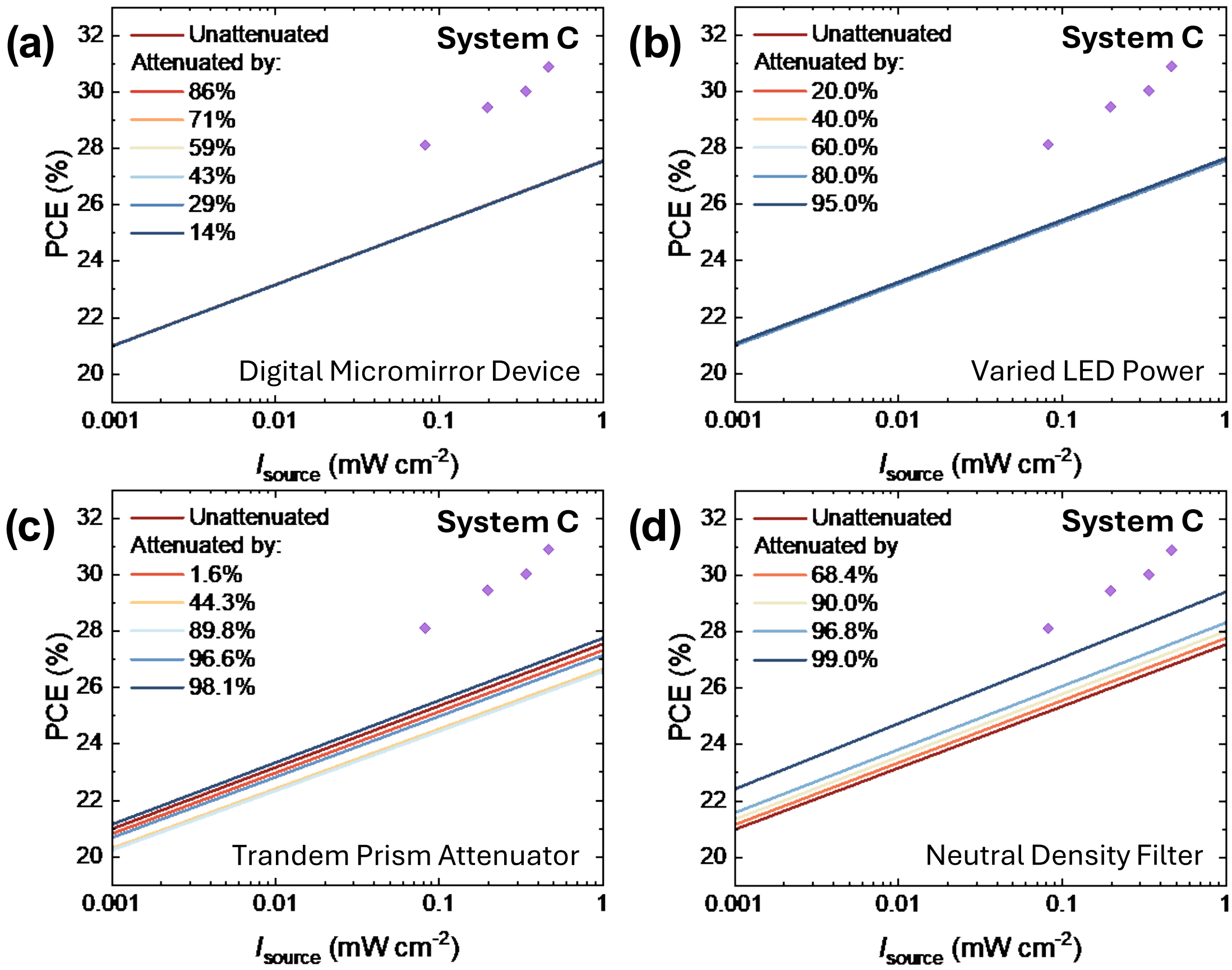

Figure S3: Power conversion efficiency (PCE) simulated using the photovoltaic external quantum efficiency spectrum and non-radiative open-circuit voltage loss of System C then plotted versus irradiance for four different attenuation techniques; namely these techniques are **(a)** a digital micromirror device, **(b)** varied LED driving powers, **(c)** a tandem prism attenuator, and **(d)** a neutral density filter. In all panels, the purple diamonds indicate the experimentally-determined measurements.

Supporting Note 3: Maximum Spatial Non-Uniformity

As discussed in **Section 2.5** of the main text, under illumination by a point source a distance h away, the spatial non-uniformity (SNU) in the irradiance (P_{source}) across a photovoltaic device-under-test (DUT) of side-length w can be written as

$$\mathrm{SNU} = \frac{P_{\mathrm{source}}(0) - P_{\mathrm{source}}(r)}{P_{\mathrm{source}}(0) + P_{\mathrm{source}}(r)} = \frac{1}{1 + 2\left(\frac{h}{r}\right)^2} \times 100\%. \tag{S3.1}$$

Here, r is the radial distance away from the centre of the DUT, which is assumed to be perfectly aligned with the light source. The maximum SNU will occur at the points furthest from the centre of the device, which for a square device is the corners. Consequently, the maximum distance from the centre is $r_{\mathrm{max}} = \sqrt{\left(\frac{w}{2}\right)^2 + \left(\frac{w}{2}\right)^2} = \frac{w\sqrt{2}}{2}$, and correspondingly the SNU peaks at

$$\mathrm{SNU}(r_{\mathrm{max}}) = \frac{1}{1 + 4\left(\frac{h}{w}\right)^2} \times 100\%. \tag{S3.2}$$

Hence, as the ratio $\frac{h}{w}$ is increased, the maximum SNU decreases. This behaviour is illustrated in **Figure S4**, where the SNU drops by three orders of magnitude from $h \sim w$ to $h \sim 10w$; however, beyond this the decrease in SNU is more gradual, and a much larger h/w is required to achieve SNUs below 0.1%. In practice, this could be achieved using focussing optics.

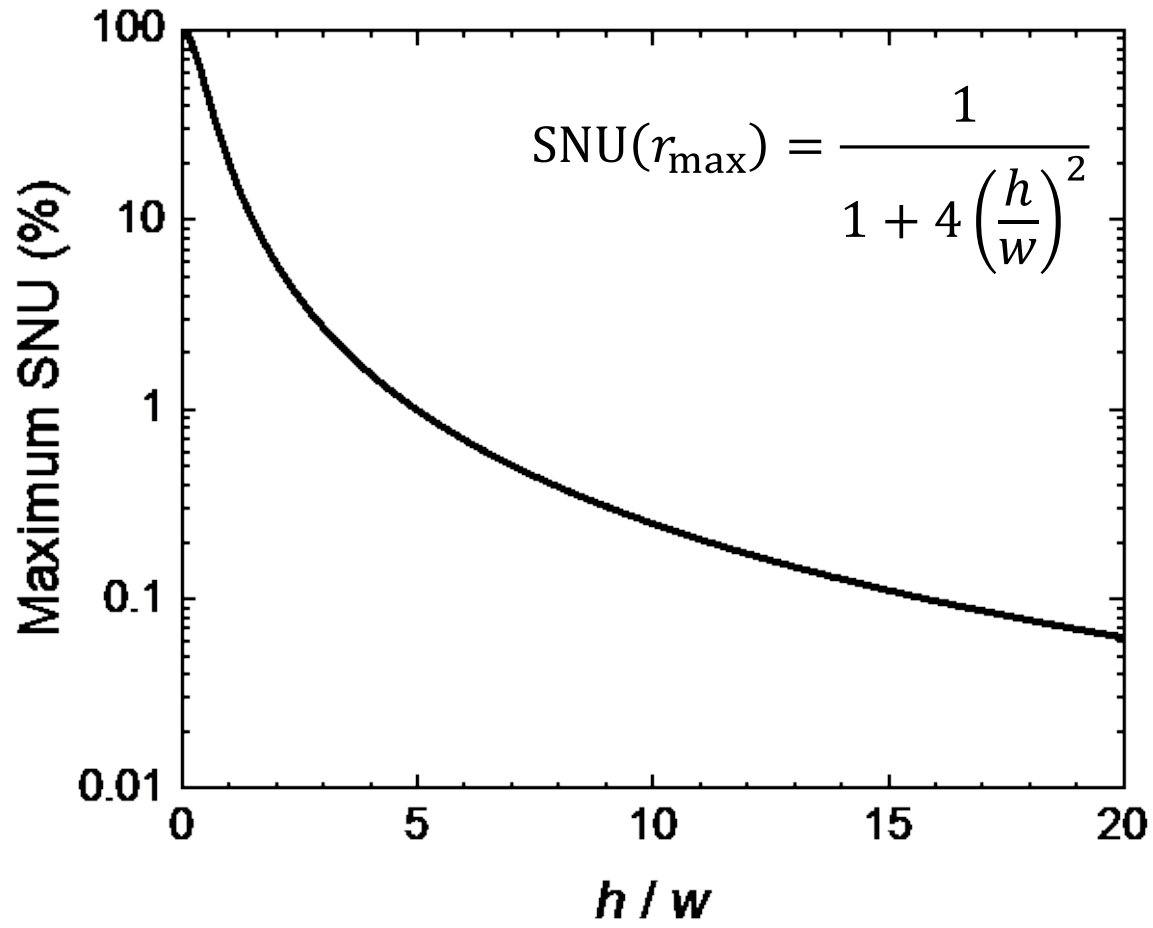

Figure S4: Maximum spatial non-uniformity (SNU) in the irradiance at the extremities of a square photovoltaic device-under-test (DUT) of side-length w, which have been calculated for various heights (h) of the point light source using Equation **(S3.2)**.

Supporting Note 4: Parameterising Diffuse Light

At a given photon energy (E), the total spectral irradiance (I_{source}) impinging on a surface element dS of photovoltaic material is the combined sum of the spectral irradiance across all incident angles. In the case of collimated light, all light impinges at normal incidence; in the perfectly-diffuse limit, on the other hand, light approaches equally across all incident angles. Consequently, with the light impinging at general angle θ (measured from normal) and azimuthal angle ϕ being descried with some angular spectral irradiance (i_{source}, in units of $\text{Wm}^{-2}\text{eV}^{-1}\text{st}^{-1}$ or similar), it must be the case that that integral of i_{source} over the whole hemisphere above dS satisfies

$$I_{\text{source}}(E) \equiv \iint_{\text{Hemisphere}} i_{\text{source}}(E, \theta, \phi)\, \text{d}\Omega, \tag{S4.1}$$

where $\text{d}\Omega =$ refers to an element of solid angle. As a hemispherical symmetry is being considered, the angles have domains $\theta \in \left[0, \frac{\pi}{2}\right]$ and $\phi \in [0, 2\pi)$. Using these limits, Equation **(S4.1)** becomes

$$I_{\text{source}}(E) \equiv \int_0^{2\pi} \int_0^{\frac{\pi}{2}} i_{\text{source}}(E, \theta, \phi) \cdot \sin(\theta)\, \text{d}\theta \text{d}\phi. \tag{S4.2}$$

To progress further, the exact nature of the angular spectral irradiance must be known. In this work, a Gaussian distribution of angular irradiance was considered, where most light impinges at the mean angle θ_0, while the amount of light impinging at alternative angles is determined by the standard deviation (σ) of this distribution. In other words, the angular spectral irradiance was modelled with a circular symmetry about the azimuth (i.e., it is independent of ϕ) and was described with

$$i_{\text{source}}(E, \theta, \phi) = A(E) \exp\left(-\frac{[\theta - \theta_0]^2}{2\sigma^2}\right). \tag{S4.3}$$

Here, $A(E)$ is a normalisation factor pinned by Equation **(S4.2)**. As the Gaussian distribution may not tend to nought for angles approaching grazing incidence ($\theta \to \frac{\pi}{2} = 90°$), then appropriate normalisation is required to ensure that an integral of i_{source} over the full hemispherical solid angle yields $I_{\text{source}}(E)$.

By combining Equations **(S4.2)** and **(S4.3)** and evaluating the integral with respect to the azimuthal angle, one finds that

$$\frac{I_{\text{source}}(E)}{2\pi A(E)} = \int_0^{\frac{\pi}{2}} \sin(\theta) \exp\left(-\frac{[\theta - \theta_0]^2}{2\sigma^2}\right) \text{d}\theta. \tag{S4.4}$$

In its current form, this integral cannot be readily evaluated. First, a variable substitution with $x = (\theta - \theta_0)/\sigma\sqrt{2}$ allows Equation **(S4.4)** to be rewritten as

$$\frac{I_{\text{source}}(E)}{2\pi A(E)} = \sigma\sqrt{2} \int_F^G \sin\left(\theta_0 + \sigma\sqrt{2}x\right) e^{-x^2}\, \text{d}x. \tag{S4.5}$$

where $F = \frac{-\theta_0}{\sigma\sqrt{2}}$ and $G = \frac{\pi}{\sigma 2\sqrt{2}} - \frac{\theta_0}{\sigma\sqrt{2}} = F + \frac{\pi}{\sigma 2\sqrt{2}}$. By making use of the sine addition formula, $\sin(A \pm B) = \sin(A)\cos(B) \pm \cos(A)\sin(B)$, Equation **(S4.5)** can be rewritten as

$$\frac{I_{\text{source}}(E)}{2\pi A(E)} = \sigma\sqrt{2}\sin(\theta_0)\int_F^G \cos\left(\sigma\sqrt{2}x\right) e^{-x^2}\,\mathrm{d}x + \sigma\sqrt{2}\cos(\theta_0)\int_F^G \sin\left(\sigma\sqrt{2}x\right) e^{-x^2}\,\mathrm{d}x. \tag{S4.6}$$

Recalling Euler's formula, $e^{ix} = \cos(x) + i\sin(x)$, it is clear that the two integrals remaining in this equation are actually the real and imaginary components of a single integral $I_\sigma = \int_F^G e^{i\sigma\sqrt{2}x} \cdot e^{-x^2}\,\mathrm{d}x$. To evaluate this integral, the square must first be completed on the exponent to write

$$I_\sigma = \exp\left(-\frac{\sigma^2}{2}\right)\int_F^G \exp\left(-\left[x - \frac{i\sigma\sqrt{2}}{2}\right]^2\right)\mathrm{d}x. \tag{S4.7}$$

A change in variables $v = x - \frac{i\sigma\sqrt{2}}{2}$ yields

$$I_\sigma = \exp\left(-\frac{\sigma^2}{2}\right)\int_{F-\frac{i\sigma\sqrt{2}}{2}}^{G-\frac{i\sigma\sqrt{2}}{2}} e^{-v^2}\,\mathrm{d}v. \tag{S4.8}$$

By making use of the definition and the odd parity of the error function,[9] $\mathrm{erf}(z) = \frac{2}{\sqrt{\pi}}\int_0^z e^{-t^2}\,\mathrm{d}t = -\,\mathrm{erf}(-z)$, then the integral can be evaluated to yield

$$I_\sigma = \frac{\sqrt{\pi}}{2}\exp\left(-\frac{\sigma^2}{2}\right)\left\{\mathrm{erf}\left(\frac{\pi - 2\theta_0 - i2\sigma^2}{2\sigma\sqrt{2}}\right) + \mathrm{erf}\left(\frac{2\theta_0 + i2\sigma^2}{2\sigma\sqrt{2}}\right)\right\}. \tag{S4.9}$$

By combining this with Equation **(S4.6)**, the normalisation factor can be expressed in terms of arbitrary σ and θ_0 (in units of radians) as

$$A(E) = \frac{1}{\sqrt{2\pi^3\sigma^2}\exp\left(-\frac{\sigma^2}{2}\right)}\frac{I_{\text{source}}(E)}{\sin(\theta_0)\,\mathbf{Re}[F_\sigma] + \cos(\theta_0)\,\mathbf{Im}[F_\sigma]}, \tag{S4.10}$$

where

$$F_\sigma \equiv \frac{2I_\sigma}{\sqrt{\pi}\exp\left(-\frac{\sigma^2}{2}\right)} = \mathrm{erf}\left(\frac{\pi - 2\theta_0 - i2\sigma^2}{2\sigma\sqrt{2}}\right) + \mathrm{erf}\left(\frac{2\theta_0 + i2\sigma^2}{2\sigma\sqrt{2}}\right). \tag{S4.11}$$

In the special case of a distribution centred about $\theta_0 = 0$, the $\sin(\theta_0)$ term vanishes from Equation **(S4.10)**, such that the angular distribution of light can be modelled with

$$i_{\text{source}}(E,\theta) = \frac{1}{\sqrt{2\pi^3\sigma^2}}\frac{I_{\text{source}}(E)}{\mathbf{Im}\,[F_\sigma]}\exp\left(\frac{\sigma^4 - \theta^2}{2\sigma^2}\right). \tag{S4.12}$$

Supporting Note 5: Angular-Dependent EQE_{PV} Measurements

Angular dependent EQE_{PV} measurements were performed on a custom-built EQE_{PV} apparatus based on state-of-the-art designs.[10] The light source used in the apparatus was a *SuperK FIANIUM* FIU15 supercontinuum laser fibre coupled into a *Photon etc*. laser line tuneable filters (LLTF) contrast. The output was modulated by an optical chopper and directed to the device-under-test (DUT), which was held on a manual rotation stage (*Thorlabs* RP01). The resulting current was amplified with a pre-amplifier (*FEMTO* DLPCA-200) and detected by a lock-in amplifier (*SRS* 860) locked to the frequency of the optical chopper. The angle of each measurement was controlled manually, while the remaining equipment was controlled via a custom-built *LabView* environment.

For both the Reference Cell and the organic (Epishine) Cell considered in **Section 2.6** main text, EQE_{PV} spectra were measured at 5° increments in incident angle. For the sake of clarity, however, only 10° increments were shown in **Figure 6c** and **6d** in the main text. To complement that figure, the omitted EQE_{PV} spectra measured for the two devices at incident angles of 5°, 15°, …, 85° are shown in **Figure S5**. For comparison, the EQE_{PV} spectra shown in the original figure are duplicated therein.

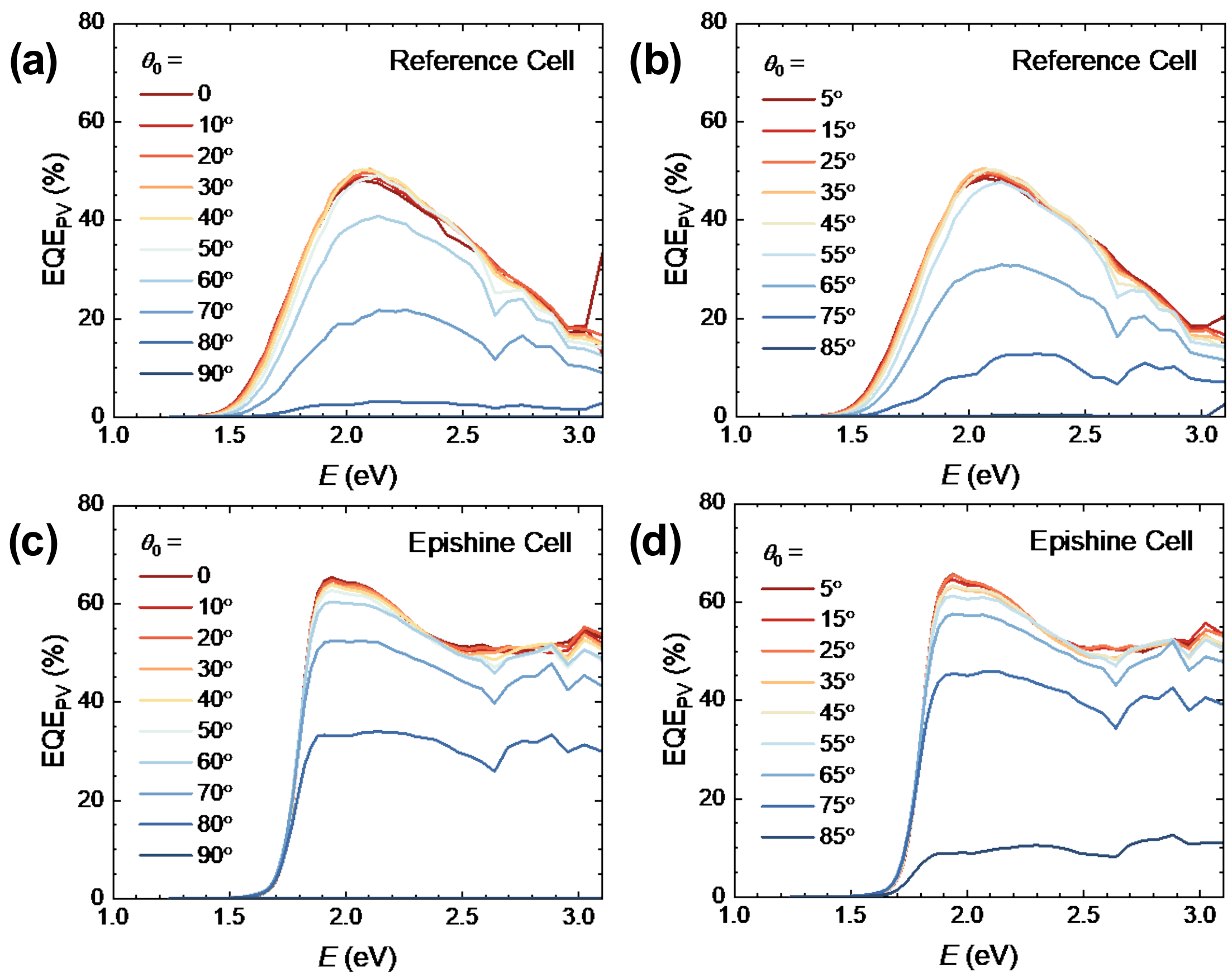

Figure S5: Angular-dependent photovoltaic external quantum efficiency (EQE_{PV}) spectra plotted versus photon energy (E) for the inorganic Reference Cell and the organic Epishine Cell considered in **Section**

2.6 of the main text. For the Reference Cell, the spectra measured for incident angles $\theta_0 = 0, 10°, \ldots,$ $80°$ are plotted in **(a)**, while the spectra measured for incident angles $\theta_0 = 5°, 15°, \ldots, 85°$ are plotted in **(b)**. Similarly, for the Epishine Cell, the spectra measured for incident angles $\theta_0 = 0, 10°, \ldots, 80°$ are plotted in **(c)**, while the spectra measured for incident angles $\theta_0 = 5°, 15°, \ldots, 85°$ are plotted in **(d)**. Note that $\mathrm{EQE_{PV}} \rightarrow 0$ has been assumed for both cells in the limit of grazing incidence (where $\theta_0 \rightarrow 90°$).

Supporting Note 6: Validated Literature Data (in Tabulated Form)

Table S1: Summary of the validated performance of indoor photovoltaics based on perovskite semiconductors. Note that only the thermodynamically-valid systems from **Figure 7** of the main text are included in this table. References to systems that are either thermodynamically implausible or lie within the "Zone of Plausibility" have intentionally been omitted to preserve the anonymity of the authors of those works. References to these systems will be made available upon request from the Corresponding Authors.

E_g (eV)	Light Source	Light Intensity	Intensity Unit	PCE (%)	V_{oc} (V)	J_{sc} (μA cm^{-2})†	FF (%)	Ref.
1.54	AM1.5G	100	mWcm^{-2}	24.6	1.19	25.03	82.4	[11]
	3000 K LED	0.056	mWcm^{-2}	37.7	1.04	284.21	73.5	
1.55	AM1.5G	100	mWcm^{-2}	20.6	1.09	24.25	79.2	[12]
	2700 K LED	0.0711	mWcm^{-2}	29.1	0.98	29.05	79.4	
1.55	AM1.5G	100	mWcm^{-2}	19.2	1.05	25.9	70.5	[13]
	Cool White LED	0.2851	mWcm^{-2}	25.5	0.82	109.1	80.6	
		0.0285	mWcm^{-2}	23.5	0.76	11.1	29.5	
		0.1425	mWcm^{-2}	24.7	0.80	54.7	80.2	
1.66	AM1.5G	100	mWcm^{-2}	19.1	1.22	20.13	74.2	[14]
	3000 K LED	0.695	mWcm^{-2}	37.5	0.98	29.05	79.4	
1.69	AM1.5G	100	mWcm^{-2}	21.3	1.21	20.83	84.6	[15]
	3000 K LED	0.3019	mWcm^{-2}	40.0	1.02	55.72	79.6	
		0.2415	mWcm^{-2}	40.3	0.98	29.28	79.1	
1.71	AM1.5G	100	mWcm^{-2}	21.3	1.21	20.83	84.6	[15]
	3000 K LED	0.3019	mWcm^{-2}	40.0	1.02	55.72	79.6	
		0.2415	mWcm^{-2}	40.3	0.98	29.28	79.1	
		0.1208	mWcm^{-2}	39.2	0.99	130	77.4	
		0.0604	mWcm^{-2}	29.4	0.88	132.2	78.6	
	2700 K FL	0.2255	mWcm^{-2}	37.0	0.91	153.3	82.5	
1.74	AM1.5G	100	mWcm^{-2}	13.7	1.08	16.6	76.2	[13]
	Cool White LED	0.0285	mWcm^{-2}	20.7	0.74	10.3	77.4	
		0.2851	mWcm^{-2}	22.5	0.81	99.5	79.1	
		0.1425	mWcm^{-2}	21.9	0.81	49.6	77.9	

†In the cases where devices are measured under AM1.5G standard testing conditions for conventional solar cells, the short-circuit current densities are quoted in units of mA cm^{-2} instead of μA cm^{-2}.

Table S2: Summary of the validated performance of indoor photovoltaics based on organic semiconductor blends. Note that only the thermodynamically-valid systems from **Figure 7** of the main text are included in this table. References to systems that are either thermodynamically implausible or lie within the "Zone of Plausibility" have intentionally been omitted to preserve the anonymity of the authors of those works. References to these systems will be made available upon request from the Corresponding Authors.

E_g (eV)	Light Source	Light Intensity	Intensity Unit	PCE (%)	V_{oc} (V)	J_{sc} (μA cm^{-2})†	FF (%)	Ref.
1.42	AM1.5G	100	$mWcm^{-2}$	15.0	0.82	25.7	71.3	[13]
	Cool White LED	0.0285	$mWcm^{-2}$	18.3	0.60	11.5	76.6	
		0.1425	$mWcm^{-2}$	19.9	0.67	54.1	77.6	
		0.2851	$mWcm^{-2}$	20.3	0.68	112.8	75.6	
1.52	AM1.5G	100	$mWcm^{-2}$	22.8	0.98	17.2	77.5	[16]
	3000 K LED	125	lux	26.6	0.80	16.8	81.4	
1.54	AM1.5G	100	$mWcm^{-2}$	15.7	0.97	22.8	71.1	[17]
	2600 K LED	0.318	$mWcm^{-2}$	25.9	0.83	131.9	75.4	
		0.509	$mWcm^{-2}$	26.4	0.84	210.6	75.7	
		0.637	$mWcm^{-2}$	27.1	0.86	263.8	76.1	
		0.159	$mWcm^{-2}$	24.4	0.81	64.4	74.7	
1.55	AM1.5G	100	$mWcm^{-2}$	11.6	0.98	18.8	63.0	[17]
	2600 K LED	0.637	$mWcm^{-2}$	21.1	0.89	214.6	70.8	
		0.159	$mWcm^{-2}$	19.5	0.84	54.3	67.9	
		0.318	$mWcm^{-2}$	20.0	0.87	106.9	68.9	
		0.509	$mWcm^{-2}$	20.9	0.88	171.8	70.7	
1.71	AM1.5G	100	$mWcm^{-2}$	14.1	1.12	10.18	64.0	[18]
	3000 K LED	700	lux	24.7	0.99	79	67.0	
		290	lux	22.6	0.95	32	65.0	
1.81	AM1.5G	100	$mWcm^{-2}$	11.6	1.21	13.65	70.4	[19]
	2700 K LED	1000	lux	29.2	1.09	101.51	80.4	
		500	lux	28.4	1.06	54.78	78.2	
		200	lux	26.4	1.01	20.3	77.1	
1.82	AM1.5G	100	$mWcm^{-2}$	9.8	1.24	11.5	68.1	[20]
	6500 K LED	1000	lux	23.1	1.09	85.4	78.9	
	2700 K LED	200	lux	22.2	1.03	18.2	71.5	
1.86	AM1.5G	100	$mWcm^{-2}$	9.0	1.21	11.3	11.3	[13]
	Cool White LED	0.1425	$mWcm^{-2}$	21.3	1.03	43.2	68.2	
		0.0285	$mWcm^{-2}$	19.2	0.97	8.62	64.9	
		0.2851	$mWcm^{-2}$	21.6	1.06	82.7	69.7	

†In the cases where devices are measured under AM1.5G standard testing conditions for conventional solar cells, the short-circuit current densities are quoted in units of mA cm^{-2} instead of μA cm^{-2}.

Table S3: Summary of the validated performance of indoor photovoltaics based on inorganic semiconductors. Note that only the thermodynamically-valid systems from **Figure 7** of the main text are included in this table. References to systems that are either thermodynamically implausible or lie within the "Zone of Plausibility" have intentionally been omitted to preserve the anonymity of the authors of those works. References to these systems will be made available upon request from the Corresponding Authors.

E_g (eV)	Light Source	Light Intensity	Intensity Unit	PCE (%)	V_{oc} (V)	J_{sc} (μA cm^{-2})†	FF (%)	Ref.
1.12	AM1.5G	100	mWcm^{-2}	25.7	0.73	42.9	81.6	[13, 21]
	Cool White LED	0.2851	mWcm^{-2}	15.5	0.52	119.2	71.3	
		0.1425	mWcm^{-2}	14.0	0.50	59.7	68.2	
		0.0285	mWcm^{-2}	10.2	0.41	12.1	58.3	
1.17	AM1.5G	100	mWcm^{-2}	17.0	0.71	32.2	74.6	[13]
	Cool White LED	0.2851	mWcm^{-2}	12.5	0.52	114.3	65.5	
		0.0285	mWcm^{-2}	4.5	0.36	11.5	33.8	
		0.1425	mWcm^{-2}	10.5	0.48	56.7	58.9	
1.47	AM1.5G	100	mWcm^{-2}	15.8	0.83	28.1	67.5	[13]
	Cool White LED	0.0285	mWcm^{-2}	15.7	0.51	12.7	68.0	
		0.2851	mWcm^{-2}	18.0	0.58	113.4	77.1	
		0.1425	mWcm^{-2}	17.3	0.56	57.7	75.6	
1.83	AM1.5G	100	mWcm^{-2}	7.9	0.89	12.9	68.0	[13]
	Cool White LED	0.2851	mWcm^{-2}	16.3	0.71	89.4	73.1	
		0.0285	mWcm^{-2}	13.7	0.61	8.88	72.1	
		0.1425	mWcm^{-2}	15.6	0.69	44.6	73.0	
1.89	AM1.5G	100	mWcm^{-2}	17.2	1.49	14.3	80.9	[13]
	Cool White LED	0.0285	mWcm^{-2}	37.5	1.28	11.2	81.3	
		0.1425	mWcm^{-2}	39.9	1.33	55.6	83.0	
		0.2851	mWcm^{-2}	40.9	1.35	112.15	83.4	
1.95	AM1.5G	100	mWcm^{-2}	7.2	0.90	13.6	59.0	[22]
	6500 K LED	1000	lux	18.0	0.77	112	63.9	

†In the cases where devices are measured under AM1.5G standard testing conditions for conventional solar cells, the short-circuit current densities are quoted in units of mA cm^{-2} instead of μA cm^{-2}.

Supporting Note 7: Effect of Width of Zone of Plausibility

In the statistically unlikely case that all comparison imprecisions are at their maximum value and act in the same direction, a 17.3% "Zone of Plausibility" was defined in the main text. **Figure S6** illustrates how the width of this zone affects the classifications of the ~100 measurements in **Figure 7** of the main text. Clearly, as the measurements designated "thermodynamically valid" do not rely on the width of the Zone of Plausibility, then the share of measurements with this classification remains fixed at around 58% of all measurements considered in this work. On the other hand, as the width of the Zone of Plausibility is increased, a larger share of implausible measurements is reclassified as plausible, until eventually they all are. At the 17.3% Zone of Plausibility defined in the main text (indicated by the vertical dashed line), around 29% of the measurements are plausible, while 12% remain implausible. If more reported measurements were considered in this analysis, the changes from implausible to plausible – while still discrete – would be more gradual and could possibly have a different distribution.

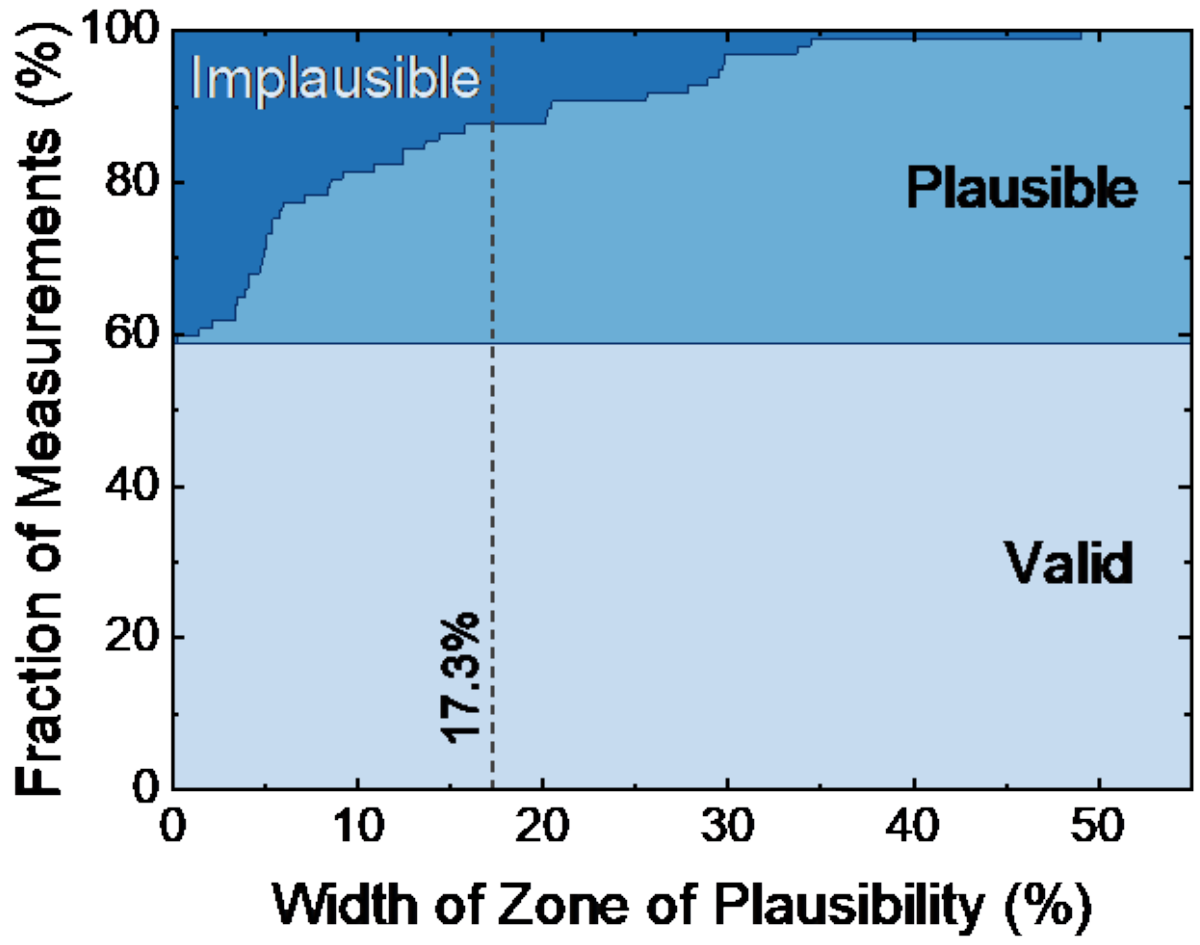

Figure S6: Fraction of total number of measurements in **Figure 7** of the main text classified as either thermodynamically "valid", "plausible", or "implausible" as the width of the Zone of Plausibility is increased. The vertical dashed line indicates the 17.3% obtained in the statistically unlikely case that all comparison imprecisions hold their maximum values and act in the same direction.

Supporting Note 8: Calibration Uncertainty for EQE_{PV} Measurements

The light intensity incident on a device-under-test (DUT) during an EQE_{PV} measurement is often calibrated using a silicon photodiode. The Newport 818-UV, UV-enhanced silicon photodiode used in our laboratory provides a representative example.[23] Its manufacturer specifies a 1% calibration uncertainty over the visible (and near-infra-red) wavelength interval 350–949 nm. Larger uncertainties are expected outside of this interval, with the Newport 818-UV's manual specifying 2% over 220–349 nm and 4% over 950–1100 nm. These figures demonstrate the typical order-of-magnitude EQE_{PV} calibration uncertainty expected for older apparatus. However, these typical values are not universal uncertainties for all EQE_{PV} measurements; in fact, the more modern Newport 818-UV/DB product listing gives 1.1% over 430–1000 nm.[24] Clearly, the uncertainty value applied to each EQE_{PV} measurement is determined by the individual detector's calibration certificate. Newport describes its detector calibration accuracies as specified at 95% confidence.[25] A 1% expanded calibration uncertainty corresponds to approximately 0.5% standard uncertainty if the certificate uses a coverage factor k = 2.

As a device's EQE_{PV} spectrum is obtained by dividing the wavelength/energy-dependent photocurrent by the incident photon flux, where this flux is determined using a calibrated reference photodiode, then EQE_{PV} is proportional to the reference responsivity. A 1% relative responsivity calibration uncertainty therefore contributes 1% relative EQE_{PV} uncertainty. Thus, an EQE_{PV} of 60% has a calibration contribution of approximately ± 0.6 percentage points. For a linear scaling of the EQE_{PV} spectrum in the ideal-diode model of **Supporting Note 1**, both photocurrent and radiative dark current scale together. With the non-radiative-loss treatment unchanged and incident irradiance fixed, the maximum-power voltage remains unchanged, whilst the calculated power conversion efficiency (PCE) scales the same as EQE_{PV}. Thus a 1% relative EQE_{PV} calibration contribution gives a 1% relative contribution to calculated PCE. For a PCE of 40.3%, this translates to approximately ± 0.40 percentage points. Wavelength-dependent errors require propagation through the spectral model.

It is important to note, however, that the photodiode calibration is one contribution to the total uncertainty. Additional contributions can arise from current-readout accuracy and noise, source drift, beam positioning, and through non-uniformities, non-linearities, temperature-dependencies, wavelength calibration, and the impact of stray light in the detector's response. Digitisation may also lead to some uncertainty. Newport explicitly distinguishes all these effects from calibration uncertainty.[25] The approximately 1% figure is therefore appropriate as a representative visible-range photodiode calibration contribution, if all other uncertainties associated with the EQE_{PV} measurement are minimal. However, this value does not establish the complete EQE_{PV} uncertainty or the uncertainty of every literature spectrum.

References

1. Kay, A.M., M.E. Fitzsimons, G. Burwell, P. Meredith, A. Armin, and O.J. Sandberg, *The Thermodynamic Limit of Indoor Photovoltaics Based on Energetically-Disordered Molecular Semiconductors*. Solar RRL, **2023**. 7(18): p. 2300277.
2. Wong, J., S.T. Omelchenko, and H.A. Atwater, *Impact of Semiconductor Band Tails and Band Filling on Photovoltaic Efficiency Limits*. ACS Energy Letters, **2020**. 6(1): p. 52-57.
3. Wurfel, P., *The Chemical Potential of Radiation*. Journal of Physics C: Solid State Physics, **1982**. 15(18): p. 3967.
4. Zarrabi, N., O.J. Sandberg, S. Zeiske, W. Li, D.B. Riley, P. Meredith, and A. Armin, *Charge-Generating Mid-Gap Trap States Define the Thermodynamic Limit of Organic Photovoltaic Devices*. Nature Communications, **2020**. 11(1): p. 5567.
5. Kay, A.M., D.B. Riley, G. Burwell, and P. Meredith, *Thermodynamic Principles for Optimizing Multi-Junction Photovoltaics—Exemplified for Perovskite-Based Indoor Photovoltaics*. APL Energy, **2025**. 3(3).
6. Rau, U., *Reciprocity Relation Between Photovoltaic Quantum Efficiency and Electroluminescent Emission of Solar Cells*. Physical Review B, **2007**. 76(8): p. 085303.
7. Kay, A.M. *Photovoltaic Performance Simulator (PV-Simulator)*. 2026; Available from: https://github.com/Austin-M-Kay/Photovoltaic_Performance_Simulator.
8. Wang, R., J. Wu, Q. Zheng, H. Deng, W. Wang, J. Chen, X. Wang, M. Wei, Z.-K. Wang, and S. Cheng, *Stable and Efficient Indoor Photovoltaics Through Novel Dual-Phase 2D Perovskite Heterostructures*. Advanced Materials, **2025**. 37(18): p. 2419573.
9. Riley, K.F. and M.P. Hobson, *Essential Mathematical Methods for the Physical Sciences*. **2011**: Cambridge University Press.
10. Zeiske, S., C. Kaiser, P. Meredith, and A. Armin, *Sensitivity of Sub-Bandgap External Quantum Efficiency Measurements of Solar Cells under Electrical and Light Bias*. ACS Photonics, **2020**. 7(1): p. 256-264.
11. Li, Y., T. Nie, X. Ren, Y. Wu, J. Zhang, P. Zhao, Y. Yao, Y. Liu, J. Feng, K. Zhao, W. Zhang, and S. Liu, *In Situ Formation of 2D Perovskite Seeding for Record-Efficiency Indoor Perovskite Photovoltaic Devices*. Advanced Materials, **2024**. 36(1): p. 2306870.
12. He, X., J. Chen, X. Ren, L. Zhang, Y. Liu, J. Feng, J. Fang, K. Zhao, and S. Liu, *40.1% Record Low-Light Solar-Cell Efficiency by Holistic Trap-Passivation using Micrometer-Thick Perovskite Film*. Advanced Materials, **2021**. 33(27): p. 2100770.
13. Müller, D., E. Jiang, P. Rivas-Lazaro, C. Baretzky, G. Loukeris, S. Bogati, S. Paetel, S.J. Irvine, O. Oklobia, S. Jones, D. Lamb, A. Richter, G. Siefer, D. Lackner, H. Helmers, C. Teixeira, D. Forgacs, M. Freitag, D. Bradford, Z. Shen, B. Zimmermann, and U. Würfel, *Indoor Photovoltaics for the Internet-of-Things–A Comparison of State-of-the-Art Devices from Different Photovoltaic Technologies*. ACS Applied Energy Materials, **2023**. 6(20): p. 10404-10414.
14. Shi, Z.-E., T.-H. Cheng, C.-Y. Lung, C.-W. Lin, C.-L. Wang, B.-H. Jiang, Y.-S. Hsiao, and C.-P. Chen, *Achieving Over 42 % Indoor Efficiency in Wide-Bandgap Perovskite Solar Cells Through Optimized Interfacial Passivation and Carrier Transport*. Chemical Engineering Journal, **2024**. 498: p. 155512.
15. Ma, Q., Y. Wang, L. Liu, P. Yang, W. He, X. Zhang, J. Zheng, M. Ma, M. Wan, Y. Yang, C. Zhang, T. Mahmoudi, S. Wu, C. Liu, Y.-B. Hahn, and Y. Mai, *One-Step Dual-Additive Passivated Wide-Bandgap Perovskites to Realize 44.72%-Efficient Indoor Photovoltaics*. Energy & Environmental Science, **2024**. 17(5): p. 1637-1644.
16. Zhou, X., H. Wu, U. Bothra, X. Chen, G. Lu, H. Zhao, C. Zhao, Q. Luo, G. Lu, K. Zhou, D. Kabra, Z. Ma, and W. Ma, *Over 31% Efficient Indoor Organic Photovoltaics Enabled by*

Simultaneously Reduced Trap-Assisted Recombination and Non-Radiative Recombination Voltage Loss. Materials Horizons, **2023**. 10(2): p. 566-575.
17. Zou, B., H.M. Ng, H. Yu, P. Ding, J. Yao, D. Chen, S.H. Pun, H. Hu, K. Ding, R. Ma, M. Qammar, W. Liu, W. Wu, J.Y.L. Lai, C. Zhao, M. Pan, L. Guo, J.E. Halpert, H. Ade, G. Li, and H. Yan, *Precisely Controlling Polymer Acceptors with Weak Intramolecular Charge Transfer Effect and Superior Coplanarity for Efficient Indoor All-Polymer Solar Cells with over 27% Efficiency*. Advanced Materials, **2024**. 36(32): p. 2405404.
18. Ma, L.-K., Y. Chen, P.C.Y. Chow, G. Zhang, J. Huang, C. Ma, J. Zhang, H. Yin, A.M. Hong Cheung, K.S. Wong, S.K. So, and H. Yan, *High-Efficiency Indoor Organic Photovoltaics with a Band-Aligned Interlayer*. Joule, **2020**. 4(7): p. 1486-1500.
19. Bi, P., S. Zhang, J. Ren, Z. Chen, Z. Zheng, Y. Cui, J. Wang, S. Wang, T. Zhang, J. Li, Y. Xu, J. Qin, C. An, W. Ma, X. Hao, and J. Hou, *A High-Performance Nonfused Wide-Bandgap Acceptor for Versatile Photovoltaic Applications*. Advanced Materials, **2022**. 34(5): p. 2108090.
20. Cui, Y., Y. Wang, J. Bergqvist, H. Yao, Y. Xu, B. Gao, C. Yang, S. Zhang, O. Inganäs, F. Gao, and J. Hou, *Wide-Gap Non-Fullerene Acceptor Enabling High-Performance Organic Photovoltaic Cells for Indoor Applications*. Nature Energy, **2019**. 4(9): p. 768-775.
21. Freunek, M., M. Freunek, and L.M. Reindl, *Maximum Efficiencies of Indoor Photovoltaic Devices*. IEEE Journal of Photovoltaics, **2012**. 3(1): p. 59-64.
22. Lu, W., M. Feng, Z. Li, B. Yan, S. Wang, X. Wen, X. An, S. Liu, J.-S. Hu, and D.-J. Xue, *Ordering One-Dimensional Chains Enables Efficient Selenium Photovoltaics*. Joule, **2024**. 8(5): p. 1430-1442.
23. Newport, *818 Series Photodetector Guide*. Newport Corporation.
24. Newport, *818 Series Photodetector Guide: User's Manual*. **2012**, Newport Corporation.
25. Newport. *Understanding Total Measurement Uncertainty in Power Meters and Detectors*. 2026; Available from: https://www.newport.com/n/measurement-uncertainty.
